# Expression, purification, and *in vitro* characterization of the carboxylesterase CEST-9.2 from *Caenorhabditis elegans*

**DOI:** 10.1042/BSR20253840

**Published:** 2026-01-21

**Authors:** Weijie Xu, Subhradeep Bhar, Steven D. Bruner, Rebecca A. Butcher

**Affiliations:** Department of Chemistry, University of Florida, Gainesville, FL, 32611, U.S.A.

**Keywords:** acyltransferase, ascaroside, biosynthesis, carboxylesterase, enzyme activity, acetylcholinesterase, pheromone, protein expression, nematode

## Abstract

The nematode *Caenorhabditis elegans* biosynthesizes the ascarosides, a large, modular family of pheromones that are used in chemical communication. A number of carboxylesterase domain-containing (CEST) enzymes are responsible for decorating the glycolipid core of the ascarosides with a variety of modifications. However, these enzymes, which are homologous to human carboxylesterases and acetylcholinesterase, have not been characterized biochemically, and thus the mechanism whereby they attach different modifications to the ascarosides is unknown. Here, we report the expression, purification, and biochemical characterization of a soluble CEST enzyme for the first time. In this study, we focused on CEST-9.2, which is responsible for making (*E*)-2-methyl-2-butenoyl (MB)-modified ascarosides. We identified candidate substrates for the enzyme, and we successfully expressed a truncated version of CEST-9.2, which is lacking the transmembrane domain, in several expression systems, including *Escherichia coli*, *Pichia pastoris*, and *Spodoptera frugiperda* Sf9 cells. The purified CEST-9.2 from each of these systems was tested against candidate substrates, including ascarosides and either MB-coenzyme A (CoA), MB-choline, or MB-carnitine. No enzymatic activity was detected using these substrates, suggesting that either the transmembrane domain is necessary for activity or that the correct substrates have not yet been identified. We showed that the purified CEST-9.2 from Sf9 cells is well-folded and dimeric, offering a potential starting point for future structural and mechanistic studies.

## Introduction

**Figure 1:**
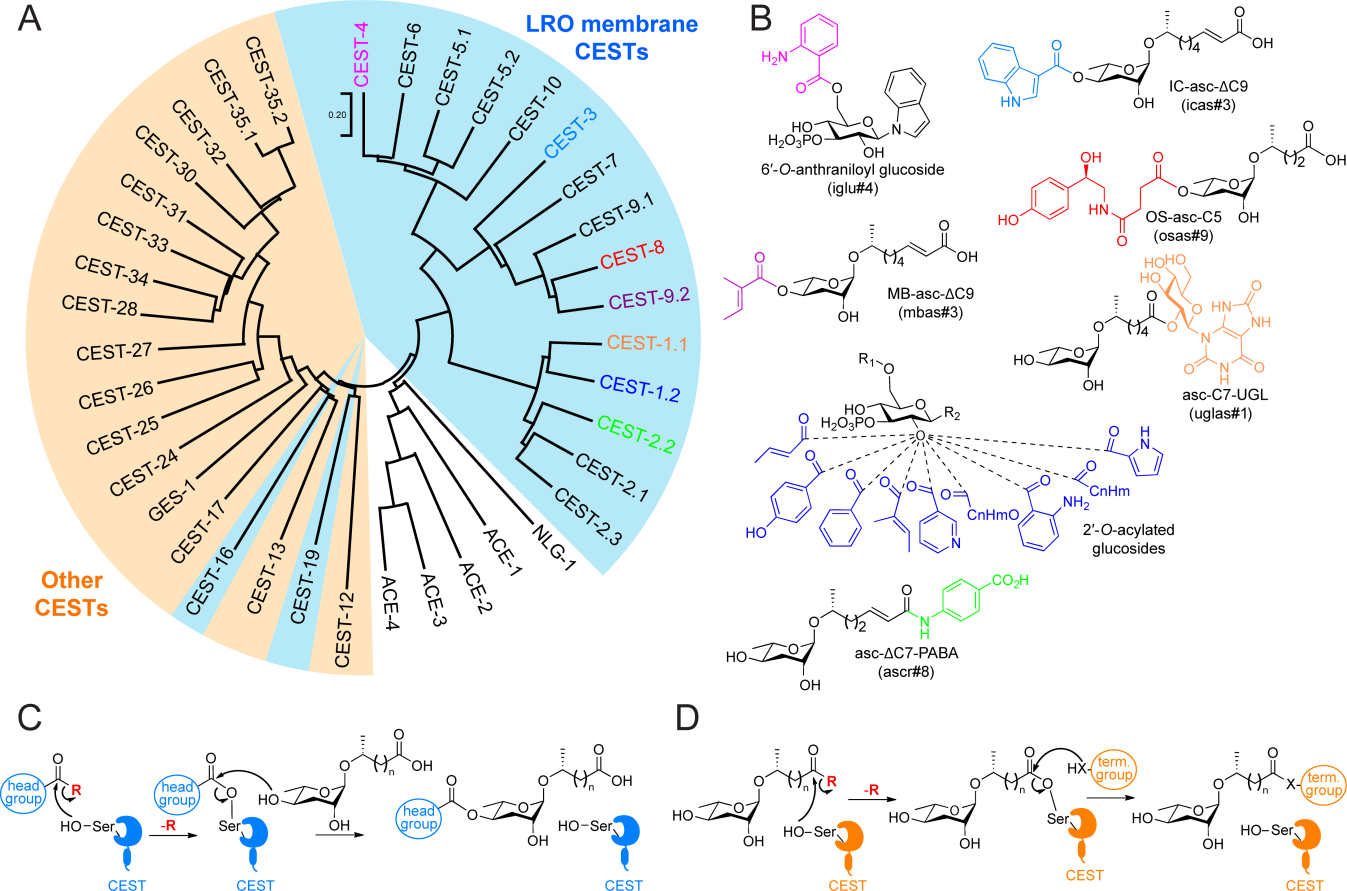
The phylogenetic tree, established roles, and proposed mechanisms of CEST enzymes. (**A**) The phylogenetic tree of CEST enzymes. The LRO membrane CEST enzymes are highlighted in light blue, and the other CEST enzymes are highlighted in light orange. *C. elegans* homologs of acetylcholinesterase (ACE-1 to -4) and neuroligin (NLG-1) are included for reference. The CEST enzymes with known biosynthetic roles are color coded according to the color of the modification groups in **Figure 1B** **B**) Examples of the modified ascarosides and glucosides that are biosynthesized by the corresponding CEST enzymes in **Figure 1A**. R_1_ represents modification groups on the 6′-position, including groups derived from various amino acid degradation products. R_2_ represents modification groups on the anomeric position, including indole, tyramine, and various others. (**C,D**) The proposed mechanisms of head-group modification (**C**) and terminus-group modification (**D**) of ascarosides. The red R group indicates an unknown activation group. In (**D**), X = O or NH.

Hundreds of ascaroside pheromones produced by nematodes have been discovered in the past two decades [1–4]. The ascarosides are built from a dideoxysugar ascarylose and a fatty acid-derived side chain [5]. This core can be modified with various building blocks derived from primary metabolites, such as amino acids, nucleosides, and sugars [5–7]. These modification groups are attached to the 2′- or 4′- position of the ascarylose as a ‘head group’ or to the fatty-acyl end of the side chain as a ‘terminus group’. The modification process for the ascarosides is carried out by a large family of over 30 carboxylesterase domain-containing (CEST) enzymes and occurs primarily in the lysosome-related organelles (LROs) (Figure 1A and B) [8–10]. The biosynthesis of modified ascarosides is also likely linked to autophagy, as the production of 4′-modified ascarosides significantly decreases in an autophagosome assembly mutant compared to wild type [9]. These discoveries suggest that *C. elegans* repurposes its degradation pathways for biosynthesizing diverse secondary metabolites and potentially links the biosynthesis of these metabolites to the metabolic status of the worms [7,11]. The diverse modifications of the ascarosides indicate a novel biosynthetic strategy for producing structurally diverse metabolites in nematodes.

A subset of the CEST enzymes have been implicated in the attachment of specific modifications to the ascarosides in *C. elegans* [8,10]. Mutant worms with loss-of-function mutations in *cest-3*, *cest-8*, and *cest-9.2* fail to make ascarosides modified with the head groups indole-3-carbonyl (IC), octopamine succinyl (OS), and MB groups, respectively [8]. Furthermore, *cest-3* mutant worms fail to attach the IC group to stable isotope-labeled ascarosides that have been exogenously provided to them[8]. Meanwhile, *cest-1.1* and *cest-2.2* mutant worms fail to make ascarosides modified with such terminus groups as uric acid gluconucleoside (UGL) and *para*-aminobenzoic acid (PABA) derivatives, respectively [10]. Additional CEST enzymes have also been implicated in the biosynthesis of various modified glucosides. The *cest-4* gene is required for the attachment of anthranilic acid to the 6′-position of certain glucosides, such as indole glucoside and *N*-acetylserotonin glucoside [7,10,12]. The *cest-1.2* gene is required for the biosynthesis of over 150 different 2′-*O*-acylated glucosides [13]. However, previous limited attempts to express the CEST enzymes have not been successful, and none of the CEST enzymes have been characterized biochemically yet [7].

*C. elegans* CEST enzymes are homologous to human carboxylesterases (CESs), acetylcholinesterase (AChE), butyrylcholinesterase (BChE), and neuroligins [8,10]. All of these enzyme families belong to the α/β-hydrolase superfamily and have a conserved Ser-His-Glu/Asp catalytic triad [14]. CESs hydrolyze a variety of endogenous esters and xenobiotics [15,16]. AChEs are a subfamily of carboxylesterases that specifically hydrolyze acetylcholine esters [17]. BChEs are highly homologous to AChEs but are non-specific cholinesterases [18]. Neuroligins are membrane proteins with an AChE-like domain [19]. Although neuroligins are closely related to AChEs structurally, they do not have a catalytic serine; instead of playing a catalytic role, they interact with other proteins on the cell surface and play a significant role in cell adhesion [20,21]. CESs, AChEs, BChEs, and neuroligins all have a signal peptide that can direct the protein into the endoplasmic reticulum (ER), where the protein can be processed, resulting in post-translational modifications such as *N*-glycosylation and disulfide bonds [15,22,23]. Neither CESs nor AChEs have a transmembrane domain, but they can be associated with membranes through different ways. CESs are associated with the membrane of the ER because they have a KDEL motif at their C-terminus, which can be recognized by KDEL receptors on the ER [15]. AChEs can be associated with the cell membrane due to protein-protein interactions with the anchoring proteins ColQ and PRiMA [24–27]. AChEs can also be anchored through modification with glycosylphosphatidylinositol [28,29]. Neuroligins are type I membrane proteins with a single transmembrane domain [30]. AChEs and BChEs can exist as monomers or form dimers and tetramers through a four-α-helix bundle near the C-terminus [31–36]. CESs can exist as monomers or form trimers and hexamers through the salt bridges across the trimer interface and constitute a trimer–hexamer equilibrium regulated by a low-affinity allosteric ligand-binding site on the surface [37,38].

CESs, AChEs, and BChEs all hydrolyze ester substrates through the conserved mechanism of the serine hydrolase family. The ester bond of the substrate is attacked by the catalytic serine, forming an acyl-enzyme intermediate that is stabilized by the oxyanion hole in the active site. The acyl-enzyme intermediate is then attacked by water to release the hydrolyzed product and free enzyme. In contrast to CESs, AChEs, and BChEs, which hydrolyze esters, CEST enzymes are predicted to have acyltransferase activity, given that they are required for the formation of ester bonds or amide bonds during the biosynthesis of modified ascarosides and glucosides [8,10]. It has been hypothesized that CEST enzymes synthesize modified ascarosides and glucosides through acyl transfer from intermediates that are activated in some way, such as CoA thioesters [10]. Notably, human CES1 possesses acyltransferase activity under certain circumstances and can transfer the acyl groups of its substrates to ethanol instead of hydrolyzing them, suggesting a balance between the hydrolysis reaction and transesterification reaction [16]. It has also been reported that a subfamily of bacterial carboxylesterases with promiscuous acyltransferase activity can transfer acyl groups to various acyl acceptors, including glucose, maltose, and aromatic alcohols, using various acyl donors, including ethyl acetate and *p*-nitrophenyl acetate [39–43].

CESs, AChEs, and BChEs from various organisms have been expressed successfully in heterologous systems. Human CES1 and rabbit CES were expressed in Sf21 insect cells [37,38]. Human AChE was expressed in HEK293 cells as well as *E. coli* cells, although the protein expressed in *E. coli* was initially insoluble and only became active after being refolded [33,44]. Rat and fish AChEs were expressed in the yeast *P. pastoris* [45,46]. One of the AChEs from *C. elegans*, ACE-1, was expressed and secreted by Sf9 insect cells [47]. Human BChE was expressed in CHO, HEK293, and S2 insect cells [48–50].

The focus of our work here was CEST-9.2 because the candidate substrates for this enzyme were commercially available or synthetically accessible. These substrates could be detected in worm extracts. Whereas full-length CEST-9.2 could not be successfully expressed in bacteria, yeast, or insect cells, a truncated CEST-9.2, in which the transmembrane domain had been removed, could be successfully expressed solubly in all three systems. The expression of the truncated protein in *E. coli* and *P. pastoris* required a maltose binding protein (MBP) tag and α-factor, respectively, to enhance solubility. Meanwhile, the expression of the truncated protein in Sf9 cells resulted in soluble protein without any additional tags to enhance solubility. Although CEST-9.2 was solubly expressed, well-folded, and dimeric, it was not active with any of the candidate substrates, indicating either that the truncated protein was not enzymatically active or that it was not provided with proper substrates or coenzymes.

## Results

### Proposing a mechanism for the CEST enzymes

Given that most CEST enzymes have a Glu-His-Ser catalytic triad and an HGGG oxyanion motif that are conserved in the CES family, we hypothesized a two-step catalytic mechanism (Figure 1C and D) [8]. For the head group-modified ascarosides, the activated head group could be attacked by the catalytic serine of the CEST enzyme to form an acyl-serine intermediate with the release of the activation group in the first step (Figure 1C). In contrast with the hydrolysis of the acyl-enzyme intermediate that is seen in CESs and AChEs, the acyl group could instead be transferred to the 4′-position of an ascaroside to form a modified ascaroside in the second step. A similar mechanism could be used by CEST enzymes to make various modified glucosides. It is unclear, however, how the substrates are activated (i.e., the identity of the red ‘R’ group in Figure 1C and D). Previously, it was hypothesized that this group was coenzyme A (CoA) or some other group. In humans, CES1 has been shown to hydrolyze fatty acyl-CoA substrates, among its many substrates, indicating that the ‘R’ group may be CoA [51]. Considering the homology between CEST enzymes and AChEs, this group could also be choline or perhaps carnitine.

For the biosynthesis of terminus group-modified ascarosides, the activated side chain of the ascaroside could be attacked by the catalytic serine, and then the ascarosyl-serine intermediate could then be attacked by the terminus group (Figure 1D). In this mechanism, the ascaroside could be activated as a CoA thioester. Alternatively, given that it has been shown that ascarosyl glucosides are up-regulated in an LRO-deficient mutant, it might suggest that the ascarosyl-CoA is first converted to an ascarosyl glucoside before reacting with a terminus group [10].

### Identification and synthesis of candidate substrates for CEST-9.2

**Figure 2:**
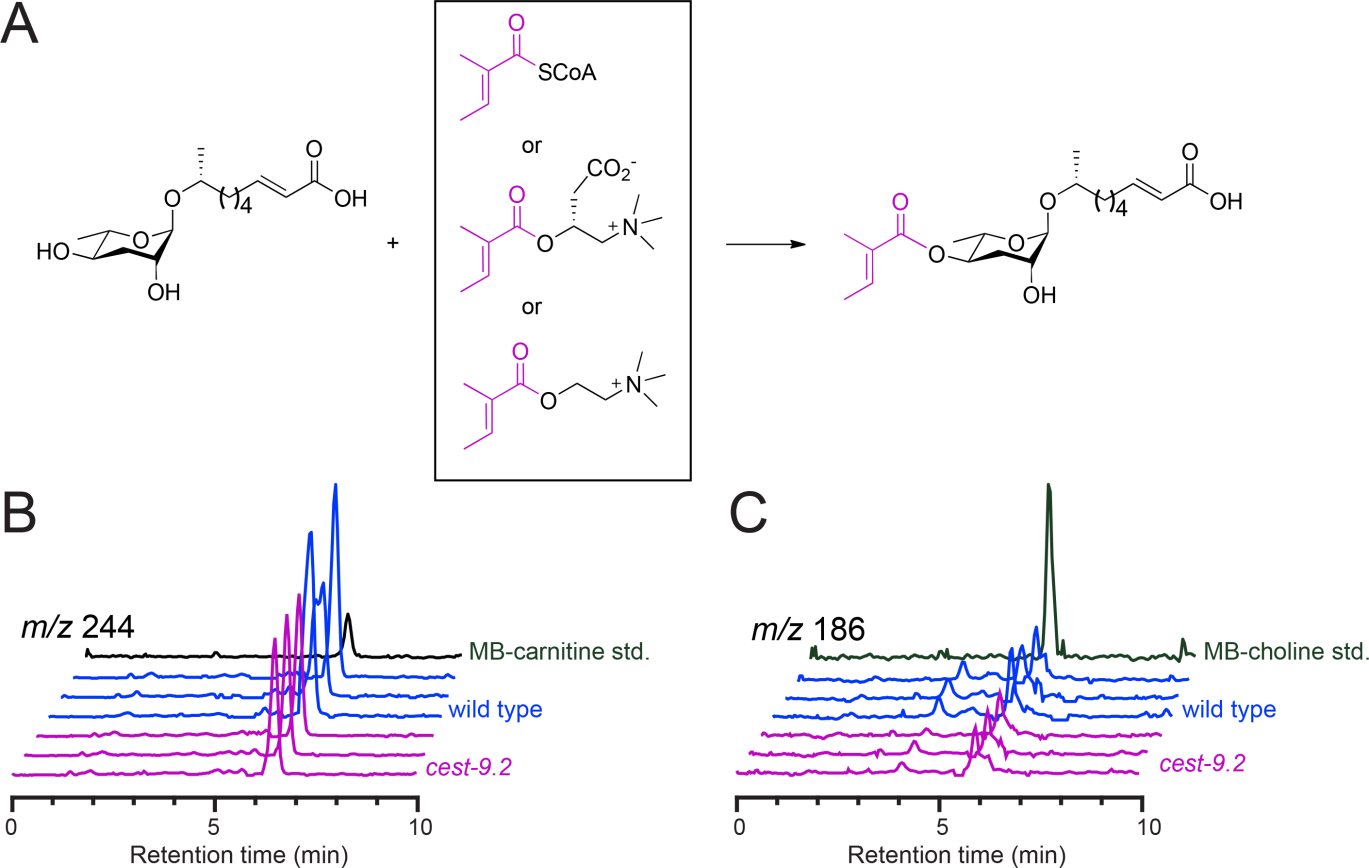
Candidate substrates of CEST-9.2 are present in *C. elegans*. (**A**) The proposed enzymatic reaction of CEST-9.2 using asc-ΔC9 and MB-CoA, MB-carnitine or MB-choline as candidate substrates. (**B,C**) The extracted ion chromatogram of MB-carnitine [M + H]^+^ (**B**) and MB-choline [M]^+^ (**C**) for the synthetic standard, wild-type worm and *cest-9.2* mutant worm samples.

Previously, we implicated CEST-9.2 in the biosynthesis of MB-ascarosides by showing that the *cest-9.2* mutant worm could not make these ascarosides [8]. Given our proposed mechanism for the CEST enzymes (Figure 1C and D), we targeted CEST-9.2 for biochemical characterization as its proposed substrates are commercially available or synthetically accessible. According to our mechanism, we could use an unmodified ascaroside (e.g., asc-ΔC9), as well as either MB-CoA, MB-carnitine, or MB-choline, as substrates (Figure 2A). MB-CoA was synthesized as described in literature (Supplementary Figure S1 and S2) [52]. MB-carnitine was commercially available. The synthesis of MB-choline has not been reported before, and we synthesized it by reacting tigloyl chloride with 2-dimethylaminoethanol, and then methylating the product using iodomethane (Supplementary Figure S3-S6). MB-CoA, or tiglyl-CoA, is a conserved intermediate in the oxidative degradation of isoleucine [53]. MB-carnitine, or tiglyl-carnitine, is a marker of β-ketothiolase deficiency, which is a defect of mitochondrial acetoacetyl-CoA thiolase in humans [54,55]. To our knowledge, however, MB-carnitine has not been described as a metabolite in *C. elegans*. Also, to our knowledge, MB-choline has not been described as a naturally occurring metabolite. However, we considered it as a possible substrate for CEST-9.2, given the homology between the CEST enzymes and AChE.

To determine whether we could detect MB-CoA, MB-carnitine, or MB-choline in *C. elegans*, we generated extracts from wild-type and *cest-9.2* mutant worms and analyzed them using LC-MS. Although the MB-CoA synthetic standard could be detected, MB-CoA could not be detected in the worm extract samples. As MB-CoA is a labile metabolite, it likely degraded during the extraction process and thus could not be detected. Surprisingly, both MB-carnitine and MB-choline were detected in wild-type worms (Figure 2B and C). However, these compounds were not more abundant in the *cest-9.2* mutant, as might occur if they were the substrates of CEST-9.2.

### Bioinformatic characterization of the CEST family

Before designing constructs for the expression of CEST-9.2, we first analyzed the CEST family of enzymes in more detail. First, we determined the closest human homolog for all the CEST enzymes by BLASTing the amino acid sequences of the CEST enzymes against human proteins. Most CEST enzymes from *C. elegans* are most closely related to one of the five CES enzymes (CES1, 2, 3, 4A, or 5A), AChE or BChE (Table 1). CEST-9.2 is most similar to CES4A, which is reported to detoxify drugs and xenobiotics in neural and cerebrospinal fluids [56]. We next analyzed the sequences of the CEST enzymes using SignalP 6.0 to determine whether they contained an N-terminal signal peptide that sorts the protein into the ER [57]. Most of the CEST enzymes, including CEST-9.2, contained this signal peptide (Table 1 **,** Supplementary Figure S 7A). Finally, we used TMHMM 2.0 to identify which CEST enzymes contain a transmembrane domain, and we used DeepLoc 2.0 to predict the subcellular localization of the CEST enzymes [58,59 ]. All of the CEST enzymes thus far linked to the biosynthesis of specific ascarosides and glucosides, including CEST-9.2, have a transmembrane domain at the C-terminus and are predicted to be localized to the LROs (Figure 1A, Table 1 **,** Supplementary Figure S7B).

**Table 1:** Amino acid sequence analysis of the CEST enzymes

Enzyme	Signal peptide	TM domain	Predicted localization	Best human homolog^a^
CEST-4	1–26	541–562	Lysosome	CES1
CEST-6	1–23	536–557	Lysosome	CES1
CEST-5.2	1–21	542–558	Lysosome	CES5A
CEST-5.1	1–18	537–557	Lysosome	BChE
CEST-10	1–21	533–553	Lysosome	CES1
CEST-3	1–20	547–567	Lysosome	BChE
CEST-7	1–36	573–593	Lysosome	NLG4
CEST-9.1	1–17	581–601	Lysosome	CES5A
CEST-8	1–17	572–592	Lysosome	CES4A
CEST-9.2	1–20	580–604	Lysosome	CES4A
CEST-1.1	1–16	636–660	Lysosome	CES2
CEST-1.2	1–18	641–662	Lysosome	AChE
CEST-2.2	1–17	637–655	Lysosome	CES2
CEST-2.1	1–18	640–661	Lysosome	CES2
CEST-2.3	1–17	630–650	Lysosome	AChE
CEST-12	1–21	n/a	Extracellular	BChE
CEST-19	1–19	574–596	Lysosome	NLG4
CEST-13	1–19	n/a	Extracellular	CES5A
CEST-16	1–17	655–677	Lysosome	AChE
CEST-17	1–18	n/a	Endoplasmic reticulum	CES2
GES-1	1–16	n/a	Endoplasmic reticulum	CES1
CEST-24	n/a	n/a	Cytoplasm	CES4A
CEST-25	n/a	n/a	Cytoplasm	CES1
CEST-26	n/a	n/a	Cytoplasm	NLG3
CEST-27	n/a	n/a	Cytoplasm	BChE
CEST-28	n/a	n/a	Cytoplasm	BChE
CEST-34	n/a	n/a	Cytoplasm	BChE
CEST-33	n/a	n/a	Peroxisome	BChE
CEST-31	n/a	n/a	Cytoplasm	BChE
CEST-30	1–19	n/a	Extracellular	BChE
CEST-32	n/a	n/a	Peroxisome	CES5A
CEST-35.1	n/a	n/a	Cytoplasm	CES5A
CEST-35.2	n/a	n/a	Cytoplasm	CES5A

^a^ The best homologs were selected based on the lowest E-value among all BLAST hits.

**Figure 3:**
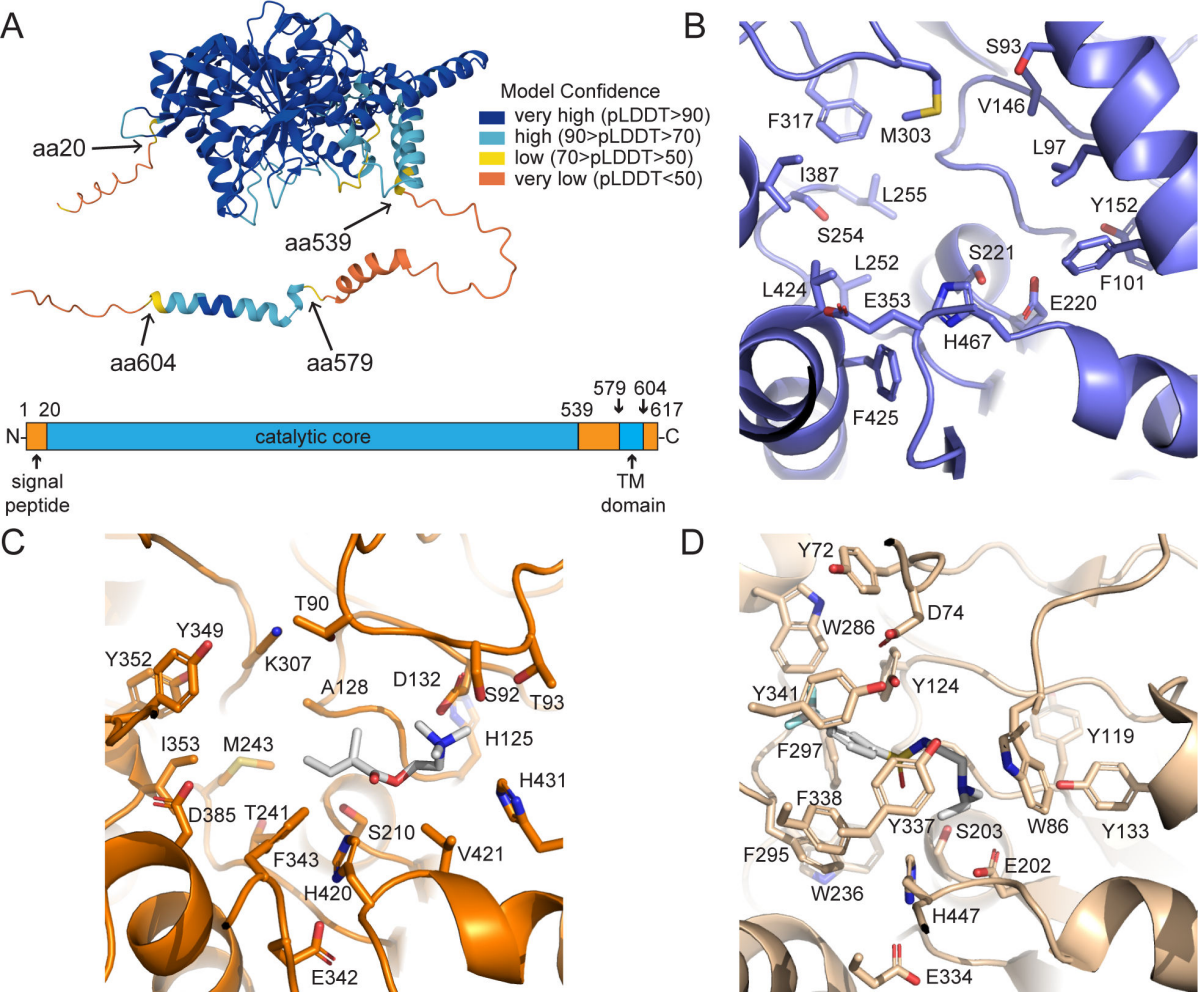
Structural analysis of the AlphaFold model of CEST-9.2. (**A**) CEST-9.2 has an N-terminal signal peptide, an unstructured region (residues 539-579) that has low local confidence in AlphaFold, and a C-terminal transmembrane domain (residues 580-604). (**B**) The active site of the crystal structure of rCES (PDB ID: 1K4Y), the protein most homologous to CEST-9.2 that has a crystal structure. (**C**) The AlphaFold model of CEST-9.2 with a candidate substrate MB-choline (white) docked in the catalytic site using AutoDock Vina. (**D**) The active site of the crystal structure of rat AChE (PDB ID: 4B84), the AChE most homologous to CEST-9.2, with a bound inhibitor (white).

The N-terminal signal peptide of CEST-9.2 extends from residues 1 to 20, and the transmembrane α-helix extends from residues 580 to 604 (Figure 3A, Table 1, Supplementary Figure S7). We attempted to express the full-length CEST-9.2 protein in this work, as well as truncated versions of the protein, lacking the transmembrane domain. In the AlphaFold model of CEST-9.2, there is a largely unstructured stretch of amino acids between the main structured portion of the protein and the C-terminal transmembrane domain (Figure 3A) [60]. Thus, to identify truncated versions of CEST-9.2 that are solubly expressed, we made constructs truncated either before the unstructured sequence (shorter truncated version) or after the unstructured sequence and before the transmembrane domain (longer truncated version).

The protein with a crystal structure that is most homologous to CEST-9.2 is rabbit liver carboxylesterase (rCES, PDB ID: 1K4Y) [38]. Similar to rCES and other CES enzymes, CEST 9.2 has a catalytic triad that consists of Ser 210, Glu342, and His420 (Figure 3B and C). The active site of CEST-9.2 is split into a large flexible pocket and a small rigid pocket by the catalytic serine (Figure 3C) [38,61]. The large pocket could potentially enable the enzyme to bind structurally different substrates, while the small pocket defines the substrate selectivity. The small pocket of rCES is covered by a conserved α-helix consisting of hydrophobic residues including Phe101 and Leu97 (Figure 3B) [38]. However, such an α-helix is missing in the AlphaFold model of CEST-9.2; instead, the small pocket of CEST-9.2 is covered by a loop containing a negatively charged Asp132 and another loop containing His431, which may together accommodate a cation substrate (Figure 3C). Although CEST-9.2 has several residues that may bind a positively charged substrate, its active site does not show much similarity to that of its closest AChE homolog among PDB structures, which is the rat AChE (PDB: 4B84). The positively charged acetylcholine is predicted to interact with multiple aromatic residues in the active site of AChE (Figure 3D), but there are fewer aromatic residues in the CEST-9.2 active site (Figure 3C). The large pocket of CEST-9.2 is smaller than that of rCES, which could explain the substrate selectivity of CEST-9.2 for small acyl groups such as MB. Two loops near the active site in the crystal structure of rCES are not visible and could potentially cover the active site when the substrate is bound [38]. Similarly, the active site of CEST-9.2 is also open and may be closed by the unstructured regions near the C-terminus (Figure 3A).

### *In silico* docking of proposed substrates and products of CEST-9.2

We performed *in silico* docking experiments on AutoDock Vina using the proposed substrates or product and the AlphaFold model of CEST-9.2 to assess the ability of CEST-9.2 to bind these ligands (Supplementary Figure S8). We selected MB-CoA, MB-carnitine, MB-choline, and MB-asc-ΔC9 as ligands because they are either candidate substrates or the proposed product of CEST-9.2. When MB-CoA was docked into the active site of CEST-9.2, the MB moiety was far away from the catalytic serine (Supplementary Figure S8A), which argues against MB-CoA being the correct substrate. Furthermore, neither positively charged residues, such as lysine and arginine, nor a glycine-rich loop were present to interact with the diphosphate group of CoA. When MB-choline was docked into the active site of CEST-9.2, Asp132 and His431 were positioned to potentially interact favorably with the choline portion of MB-choline (Figure 3C). However, when MB-carnitine was docked into the active site of CEST-9.2, its carnitine portion was positioned near Asp132 but far away from His431 due to the steric hindrance of the carboxylate moiety (Supplementary Figure S8B). Additionally, positively charged residues that might be expected to stabilize the carboxylate moiety of MB-carnitine are not seen in the active site of CEST-9.2. Some positively charged residues such as Arg542, Arg572, and Lys575 are located in the unstructured region in the AlphaFold model of CEST-9.2 and may form a part of the active site. When these residues are ordered, they could play a role in stabilizing the negatively charged moieties in the ligands discussed above. Additionally, the active sites of acyltransferases are always located deeply inside a tunnel, while the active site in the AlphaFold model of CEST-9.2 is wide open, which suggests that the unstructured sequence may cover the active site when it is ordered. Notably, when the docking experiment was performed with MB-asc-ΔC9, the proposed product, half of such a tunnel could be seen (Supplementary Figure S8C). The other half of the tunnel was missing, although it could potentially be formed by the unstructured sequence.

### Expression of CEST-9.2 in *E. coli*

**Figure 4:**
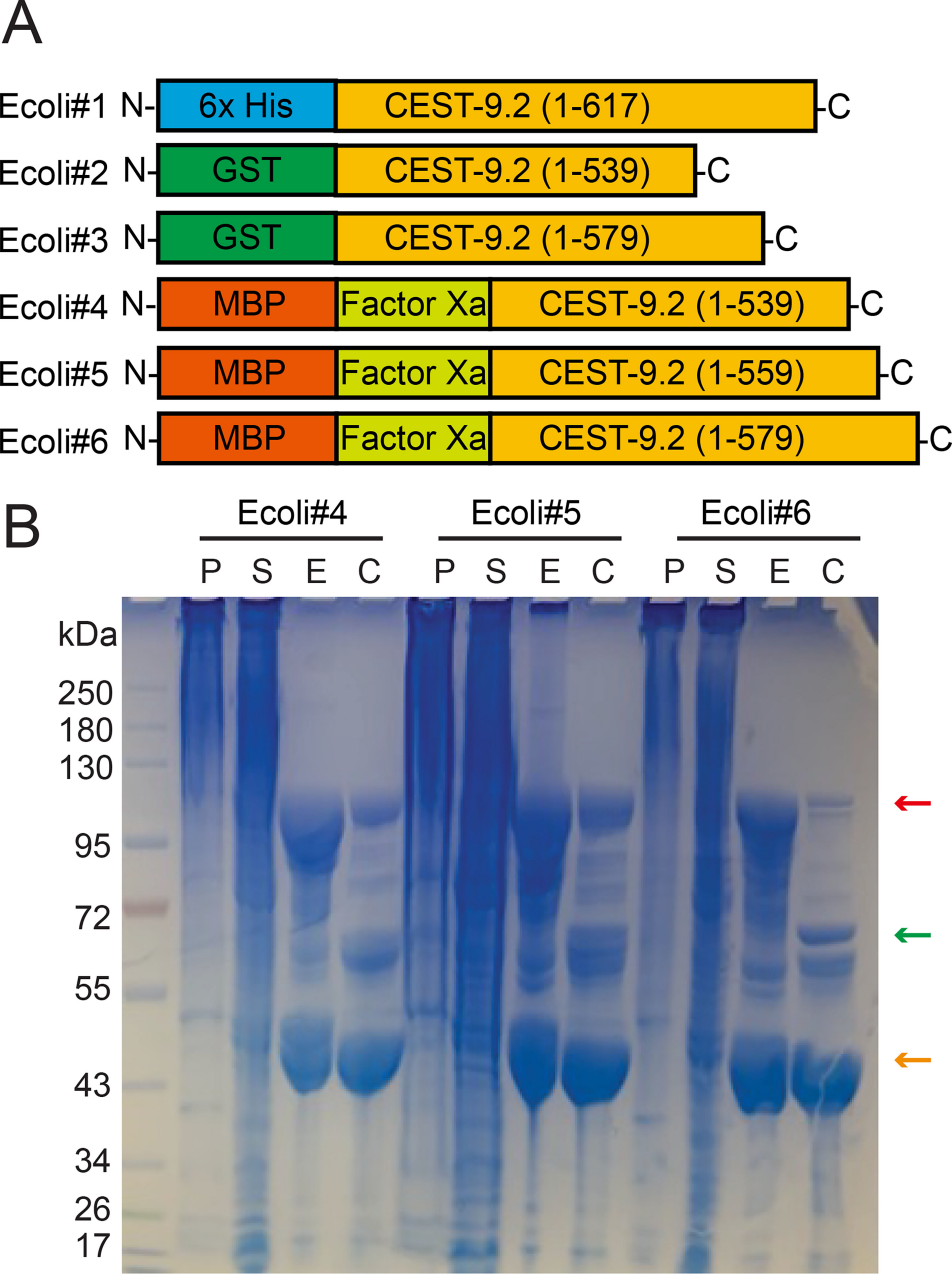
The expression of CEST-9.2 in *E. coli* . (**A**) The constructs generated in this study for expressing CEST-9.2 in *E. coli* **B**) The SDS-PAGE of all purification fractions and Factor Xa cleavage reactions of MBP-tagged CEST-9.2. P: cell pellet. S: supernatant. E: Ni-NTA elution. C: post-cleavage by Factor Xa for 3 h at room temperature. MBP-CEST-9.2 fusion protein, CEST-9.2 with MBP tag removed and free MBP tag are indicated by red, green and orange arrows, respectively.

We initially attempted to express CEST-9.2 in *E. coli*. Constructs were generated to express His-tagged, full-length CEST-9.2 and GST-tagged truncated CEST-9.2 (truncated at residue 539 or 579) (Ecoli#1-Ecoli#3, Figure 4A, Supplementary Table S1). The protein was expressed in all cases but remained in inclusion bodies (Supplementary Figure S9). Considering that CEST enzymes are predicted to have disulfide bonds, we expressed the protein in SHuffle cells, which have enhanced capacity to express correctly folded proteins with disulfide bonds in the cytoplasm, but these efforts did not improve protein solubility (Supplementary Figure S9) [62].

To improve solubility, we generated constructs to express truncated CEST-9.2 (truncated at residue 539, 559, or 579) as N-terminal MBP-tag fusions (Ecoli#4-Ecoli#6; Figure 4A, Table 2, Supplementary Table S1). The shortest version was truncated before the unstructured sequence, the medium-length version in the middle of the unstructured sequence, and the longest version before the transmembrane domain (Figure 3A). All three constructs expressed solubly (Figure 4B).

**Table 2:** CEST-9.2 constructs that have been expressed solubly

Constructs	Features	Expression systems
*E. coli* #4	CEST-9.2 (1–539) truncated before unstructured sequence N-terminal MBP tag	SHuffle T7 Express
*E. coli* #5	CEST-9.2 (1–559) truncated in the middle of the unstructured sequence N-terminal MBP tag	SHuffle T7 Express
*E. coli* #6	CEST-9.2 (1–579) truncated before transmembrane domain N-terminal MBP tag	SHuffle T7 Express
Pichia #5	CEST-9.2 (21–579) truncated before transmembrane domain N-terminal α-factor sequence C-terminal 6 x His tag	*P. pastoris* GS200
Sf9 #3	CEST-9.2 (1–579) truncated before transmembrane domain C-terminal 6 x His tag	Sf9 insect cells
Sf9 #8	CEST-9.2 (1–579) truncated before transmembrane domain C-terminal TEV site C-terminal 10 x His tag	Sf9 insect cells
Sf9 #14	CEST-9.2 (1–579) truncated before transmembrane domain (GGGS)_2_ linker between CEST-9.2 and C-terminal TEV site C-terminal Strep tag II	Sf9 insect cells

Then, we characterized the enzymatic activity of all three MBP-tagged truncated CEST-9.2 enzymes with the ascaroside asc-ΔC9 and either MB-CoA, MB-carnitine, or MB-choline as the candidate substrates (Figure 2A). We tested the activity of the MBP-tagged CEST-9.2 at either pH 5.2 or pH 7.4 but could not detect the likely product of CEST-9.2, MB-asc-ΔC9. Furthermore, removal of the MBP tag from CEST-9.2 did not result in an active enzyme.

### Expression of CEST-9.2 in *P. pastoris*

**Figure 5:**
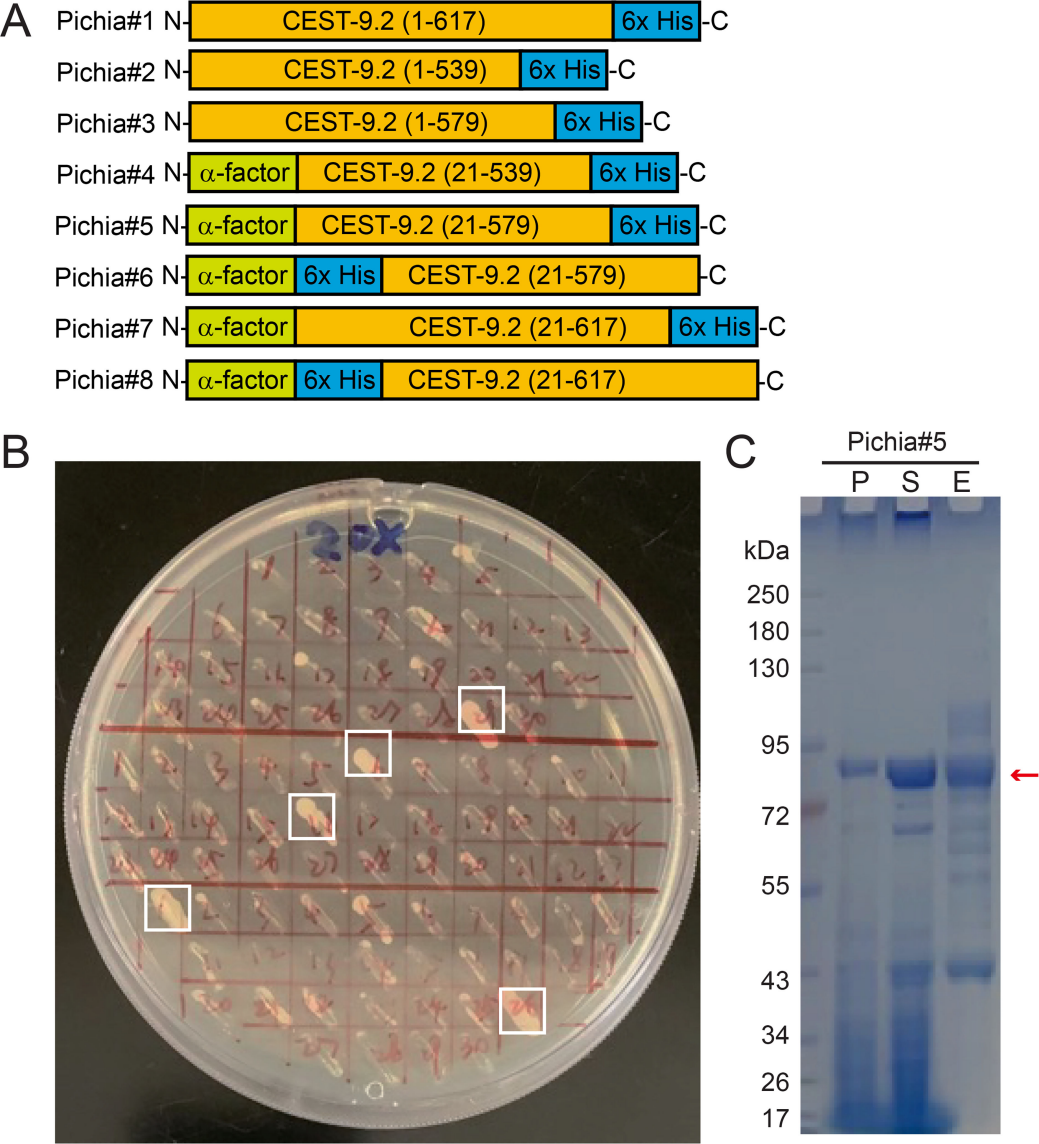
The expression of CEST-9.2 in *P. pastoris* . (**A**) The constructs generated in this study for expressing CEST-9.2 in *P. pastoris* **B**) The *P. pastoris* colonies with multiple copies of the *cest-9.2* gene (highlighted in white boxes) were selected by Zeocin. (**C**) The SDS-PAGE of all purification fractions of CEST-9.2 expressed in *P. pastoris*. P: cell pellet. S: supernatant. E: Ni-NTA elution.

Considering that CEST-9.2 has a signal peptide that directs it to the ER and it is predicted to have disulfide bonds and post-translational modifications such as *N*-glycosylation that occur in the ER (Supplementary Figure S10)[63], a eukaryotic expression system may be necessary to ensure that CEST-9.2 is correctly folded and active. First, we made several constructs for expressing full-length and truncated His-tagged CEST-9.2 using either the native signal sequence or α-factor signal sequence in yeast *P. pastoris* GS200 cells (Pichia#1 to Pichia#5; Figure 5A **,** Table 2, Supplementary Table S1). The plasmids were transformed into yeast cells by electroporation, and the colonies with multiple copies of integrated insertions in their genomic DNA were screened by being restreaked on a YPD plate containing zeocin (Figure 5B). The colonies that grew on the plate were tested for protein expression in small-scale cultures by anti-His western blot. Among these five constructs, only the longer truncated one with an N-terminal α-factor signal sequence and a C-terminal His tag (Pichia#5; Figure 5A, Table 2) was expressed as soluble protein successfully, while an analogous shorter truncated one (Pichia#4) was expressed as insoluble protein and the remaining ones with a native signal sequence (Pichia#1-Pichia#3) did not express at all. The soluble protein (Pichia#5) could be purified by Ni-NTA resin and had a molecular weight that is higher than expected (63 kDa), which suggests that the protein may be glycosylated, or that the α-factor sequence could not be cleaved post-translationally, or both (Figure 5C). We performed glycosylation analysis by using endoglycosidase H_f_ to remove the glycans [64]. The molecular weight of the protein shifted lower but was still higher than the expected molecular weight, indicating that the protein is glycosylated and that the α-factor sequence was likely not cleaved post-translationally (Supplementary Figure S 1 1).

We tested the enzymatic activity of the purified CEST-9.2 at either pH 5.2 or pH 7.4 using candidate substrates asc-ΔC9 and MB-CoA, MB-carnitine, or MB-choline, but no product was detected. Considering that the enzyme’s activity might have been inhibited by the C-terminal His tag that is close to the active site, we made another construct where we inserted the His tag between the α-factor signal sequence and CEST-9.2 (Pichia#6), but this construct was not expressed. Our attempts to express constructs that included the transmembrane domain (Pichia#7 and Pichia#8) also failed.

### Expression of CEST-9.2 in Sf9 cells

**Figure 6:**
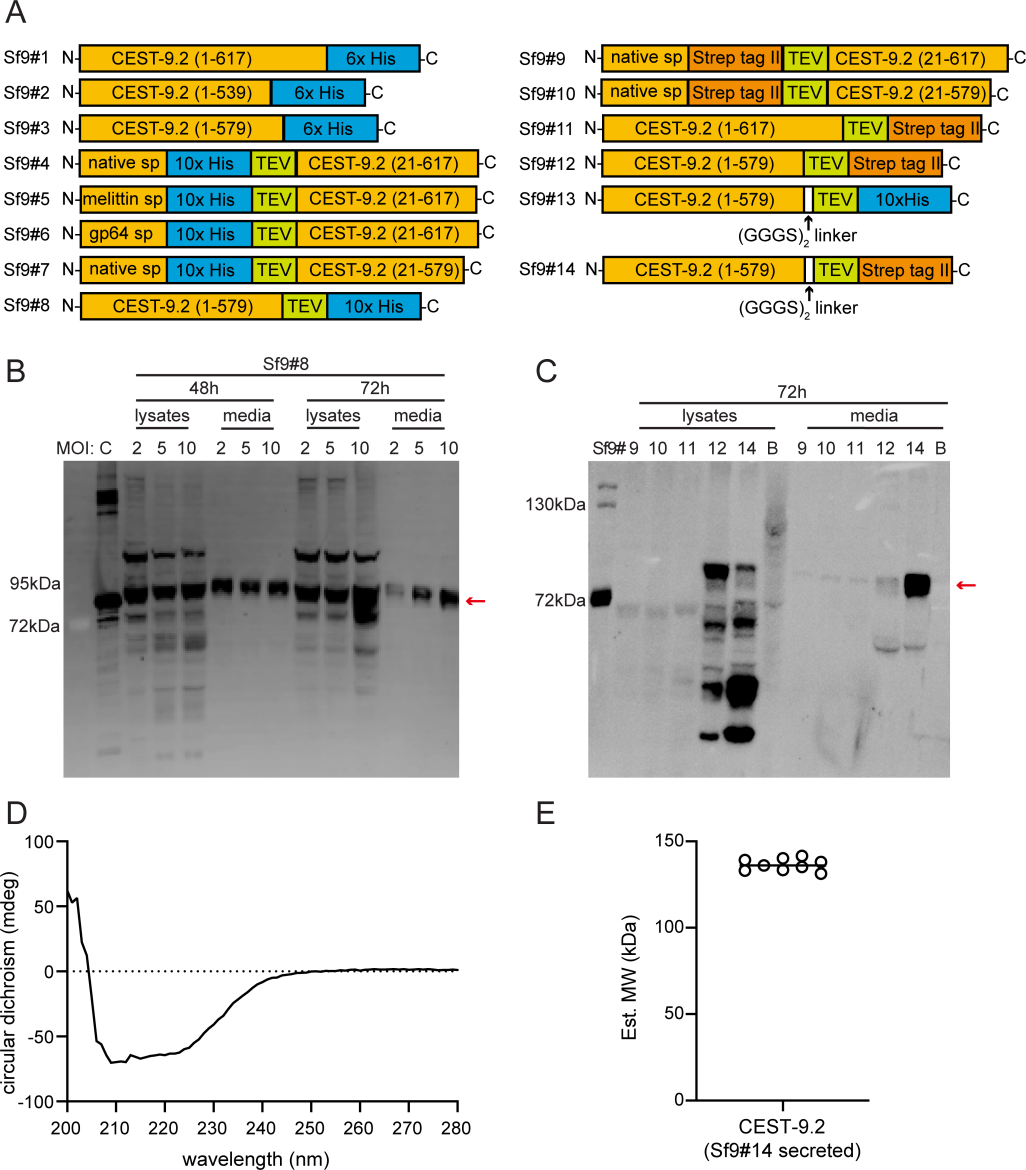
The expression of CEST-9.2 in Sf9 insect cells. (**A**) The constructs generated in this study for expressing CEST-9.2 in Sf9 insect cells. (**B**) The anti-His tag western blot of Sf9#8 . The expression of Sf9#8 was optimized by various MOIs (2, 5, or 10) and various infection times (48 h or 72 h). A His-tagged protein used as the positive control was labeled as C. (**C**) The anti-Strep tag western blot of all Strep-tagged constructs shown in **Figure 6A**. All the cells were infected at MOI of 5 for 72 h using the baculovirus for each construct. Blank control (no baculovirus infection) was used in the western blot and was labeled as B. (**D**) The CD spectrum of CEST-9.2 from Sf9#14 in TBS buffer. (**E**) The dynamic light scattering analysis of CEST-9.2 from Sf9#14. The data represent nine replicates.

It has been reported that the *C. elegans* acetylcholinesterase ACE-1 can be successfully expressed as an active protein in Sf9 insect cells [47]. Given that nematodes have an expression and post-translational modification system that is more similar to that of insect cells than that of yeast, the insect cell line Sf9 may be a better system for the expression of the CEST enzymes. We made three constructs with the native signal sequence and a C-terminal His tag, including full-length and two different truncations of CEST-9.2 (Sf9#1, Sf9#2, and Sf9#3; Figure 6A, Supplementary Table S1). The full-length CEST-9.2 (Sf9#1) was expressed but cleaved near the C-terminal transmembrane domain as only bands with very small molecular weight were observed by western blot (Supplementary Figure S12A). The shorter truncated CEST-9.2 (Sf9#2) was not expressed, while the longer truncated CEST-9.2 (Sf9#3; Table 2) was expressed intracellularly (Supplementary Figure S12B). We purified Sf9#3 and tested its activity at either pH 5.2 and pH 7.4 using asc-ΔC9 and MB-CoA, MB-carnitine, or MB-choline as candidate substrates, but the mass of expected product, MB-asc-ΔC9, was not detected by LC-MS. *p*-nitrophenyl acetate has been widely used as a general substrate for esterases with a Ser-His-Glu/Asp catalytic triad [65]. We tested whether Sf9#3 has carboxylesterase activity using a *p*-nitrophenyl acetate hydrolysis assay [66]. However, Sf9#3 could not catalyze the hydrolysis of *p*-nitrophenyl acetate at either pH 5.2 or pH 7.4. This result could mean that the enzyme is inactive, or it could mean that it excludes water from its active site. Considering that the lack of activity of Sf9#3 might be due to interference from the C-terminal His tag, we made a few more constructs with an N-terminal His tag (Sf9#4 to Sf9#7; Figure 6A, Supplementary Table S1). To test the effect of different signal peptides on protein expression level, we also replaced the native signal sequence of CEST-9.2 with either honeybee melittin signal peptide or baculovirus glycoprotein 64 (gp64) signal peptide in two of these constructs (Sf9#5 and Sf9#6; Supplementary Table S1). Unfortunately, none of these constructs could be expressed.

Concerned that the His tag at the C-terminus potentially interferes with the activity of CEST-9.2, we inserted a TEV protease site between the His tag and CEST-9.2 (Sf9#8; Figure 6A, Table 2, Supplementary Table S1). This protein was expressed and secreted to the Sf9 culture medium, and the medium collected 48 hours after infection had more protein than that collected 72 hours after infection (Figure 6B). We then attempted to cleave the His tag off using Super TEV II protease, but the cleavage reactions had very poor efficiency at either 4°C overnight or at 30°C for 1 h. We tested the activity of secreted Sf9#8 at pH 5.2 and pH 7.4 using asc-ΔC9 and MB-CoA, MB-carnitine, or MB-choline as candidate substrates, but the reactions could not produce the desired product, MB-asc-ΔC9. The *p*-nitrophenyl acetate hydrolysis assay with Sf9#8 also suggested that it does not have esterase activity (Supplementary Figure S13).

In the previous constructs, the TEV protease cleavage site may not be accessible to Super TEV II protease because the TEV site is too close to CEST-9.2. To make the TEV protease cleavage site more accessible, we made another construct (Sf9#13; Figure 6A, Supplementary Table S1), by inserting a flexible GGGS-GGGS linker between CEST-9.2 and the TEV protease cleavage site. We also replaced the His tag with Strep tag II at either the N-terminus or C-terminus in several other constructs (Sf9#9 to Sf9#12, Sf9#14; Figure 6A, Supplementary Table S1). The longer truncated version of CEST-9.2 with a C-terminal Strep tag II (Sf9#14, Figure 6A, Table 2) could be expressed and secreted to the culture medium (Figure 6C). The secreted CEST-9.2 from Sf9#14 could be purified using StrepTactin resin. Its circular dichroism (CD) spectrum suggests that it is a folded protein, and its dynamic light scattering suggests that it is dimeric (Figure 6D and E). However, the CEST-9.2 from Sf9#14 could not make MB-ascarosides using asc-ΔC9 or asc-C9 and MB-CoA, MB-carnitine, or MB-choline as candidate substrates at either pH 5.2 or pH 7.4. The CEST-9.2 from Sf9#14 could also not hydrolyze *p*-nitrophenyl acetate, which indicates the lack of hydrolase activity. Incubation of CEST-9.2 from Sf9#14 along with TEV protease at 30℃ for 1 h efficiently removed the Strep tag II (Supplementary Figure S14). However, the tag-free CEST-9.2 did not have activity using asc-ΔC9 or asc-C9 and MB-CoA, MB-carnitine, or MB-choline. Furthermore, the tag-free CEST-9.2 also did not have hydrolase activity using a *p*-nitrophenyl acetate substrate. In conclusion, the lack of activity of tag-free CEST-9.2 suggests that the true substrates of CEST-9.2 are still unknown or that the truncated version of CEST-9.2 is not active and the transmembrane domain may play an essential role in the folding or activity of functional enzyme.

## Discussion

Previous studies have revealed that CEST enzymes are required for the biosynthesis of modified ascarosides and glucosides [8,10]. However, the catalytic mechanism and the substrate scope of the CEST enzymes are unknown, and previous attempts to express CEST enzymes have failed [10]. Heterologous expression of the CEST enzymes would confirm their role in ascaroside and glucoside biosynthesis and could potentially enable the chemoenzymatic synthesis of modified ascarosides and glucosides. Here, we successfully expressed MBP-tagged truncated versions of CEST-9.2 in *E. coli*, a His-tagged truncated version of CEST-9.2 in yeast, and His-tagged and Strep-tagged truncated versions of CEST-9.2 in insect cells (Table 2). Among the many constructs we made to express truncated CEST-9.2, those which truncated the enzyme to include the unstructured region but not the transmembrane domain (that is, those truncated at residue 579) were more likely to be expressed than those that did not include either the unstructured region or the transmembrane domain (that is, those truncated at residue 539) (Figures 4A–6A, Table 2, Supplementary Table S1). Thus, this unstructured region appears to be important for expression. Although we were able to express truncated CEST-9.2, it did not have hydrolase or acyltransferase activity. The absence of activity of the truncated CEST-9.2 might be because we did not use the correct substrate. We tested various candidate substrates of CEST-9.2, including MB-CoA, MB-carnitine, and MB-choline, but potentially none of these are the true substrate of CEST-9.2.

The lack of activity may also be because the expressed enzymes are truncated. None of the constructs for expressing full-length CEST-9.2 including the transmembrane domain resulted in expression of full-length protein. For Sf9#1, a construct to express full-length CEST-9.2 with a C-terminal His tag, the protein was expressed but was cleaved near the C-terminal transmembrane domain as we could only purify a His-tagged membrane protein with a very small size in the solubilized membrane fraction. While it is not clear whether the transmembrane domain is required for the activity of the CEST enzymes, for proteins with a single-spanning transmembrane domain, sometimes the transmembrane domain is involved in regulation of the enzymatic activity [67,68]. So, it might be possible that functional CEST enzymes require the transmembrane domain, and thus, the truncated CEST-9.2 enzymes we have expressed so far are inactive. Since it is very challenging to express CEST enzymes that have post-translational modifications and a transmembrane domain, such as CEST-9.2, those CEST enzymes without a signal peptide or a transmembrane domain may be good choices for expression (Table 1).

Overall, by generating a large number of expression constructs and utilizing different heterologous systems, we have identified rules that enable successful expression of a soluble truncated form of CEST-9.2; namely, truncation of the protein after the unstructured region, but before the transmembrane domain, leads to the expression of soluble protein. Furthermore, whereas in *E. coli* and *P. pastoris*, the truncated protein is only expressed and soluble as a fusion protein (with MBP or α-factor, respectively), in Sf9 insect cells, the truncated protein is expressed and soluble on its own. We further characterized this truncated protein by CD spectroscopy and dynamic light scattering and showed that it is properly folded and likely forms a dimer, possibly through a four α-helix bundle, analogous to the dimerization mechanism of AChE. Our work is the first time that a CEST enzyme has been expressed as soluble protein.

It is not certain whether the true substrates of CEST-9.2 have been identified. In the future, it might be fruitful to use unbiased methods to screen for these substrates. For example, unbiased comparative metabolomics of wildtype versus *cest-9.2* mutant worms could be employed to determine whether the substrate(s) of the enzyme accumulate in the mutant worms. Furthermore, the truncated CEST-9.2 that was successfully expressed in Sf9 insect cells may prove useful for identifying candidate substrates in *C. elegans* extracts; that is, unbiased comparative metabolomics could be performed between *C. elegans* extracts treated without and with the truncated CEST-9.2 enzyme to determine whether the extract contains substrates for the enzyme. In addition, crystallization and structural analysis of the truncated CEST-9.2 protein with candidate substrates could be used to shed some light on the true substrates of the enzyme. Identification of these substrates would greatly extend our understanding of how CEST enzymes control the production of a vast array of ascaroside and glucoside secondary metabolites in nematodes.

## Materials and methods

### Expression and purification of His-tagged CEST-9.2 in *E. coli*

The *cest-9.2* gene was cloned from the cDNA library of *C. elegans* into pET-28a vector using the primers listed in Supplementary Table S2 and the restriction sites listed in Supplementary Table S1. The plasmid for each construct was transformed into BL21(DE3) competent cells or C41(DE3) competent cells and plated on LB-agar plates with 50 µg/ml kanamycin at 37°C overnight. A single colony was picked and inoculated into 5 ml of LB with 50 µg/ml kanamycin and grown at 37°C overnight. The next day, 5 ml starting culture was inoculated into 1 l LB culture supplemented with 50 µg/ml kanamycin. The cells were grown at 37°C, and 0.5 mM IPTG was added to induce protein expression once the OD_600_ reached 0.6. Protein expression was induced overnight at 16°C. The cells were harvested by centrifugation at 3500 rpm for 10 min using a Thermo Scientific ST40R centrifuge. The cell pellet from 2 l of culture was resuspended in 25 ml of Tris lysis buffer (25 mM Tris-HCl, pH 7.4, 500 mM NaCl) and frozen by placing in the –80°C freezer until purification.

The frozen cells were thawed and lysed through three cycles of microfluidization at 20000 psi using a Microfluidizer Processor (Microfluidics). The cell lysate was centrifuged using an Eppendorf 5810R centrifuge at 12000 rpm for 30 min at 4°C to remove cell debris and insoluble proteins. 500 µl of Ni-NTA resin was added to a column and was equilibrated with 5 ml of Tris lysis buffer. The supernatant was added to the column and incubated on ice for 1 h on a rocking platform. Then, the column was drained and washed with 5 ml Tris lysis buffer twice, followed by 5 ml Tris washing buffer (25 mM Tris-HCl, pH 7.4, 500 mM NaCl, 20 mM imidazole) twice. 5 ml of Tris elution buffer (25 mM Tris-HCl, pH 7.4, 500 mM NaCl, 250 mM imidazole) was added to the column, and the column was incubated on ice for 30 min on a rocking platform. The eluted protein was collected in a 15 ml falcon tube, and then the column was eluted again with another 5 ml of Tris elution buffer. The fractions collected in the purification process were then analyzed by SDS-PAGE. The purified protein was detected by western blot using a 6xHis tag monoclonal antibody conjugated with DyLight 488 (Invitrogen). The uncropped western blot images are shown in Supplementary Figure S15.

### Expression and purification of MBP-tagged CEST-9.2 in *E. coli*

The *cest-9.2* gene was cloned into the pMAL-c5x vector using the primers listed in Supplementary Table S2 and the restriction sites listed in Supplementary Table S1. The plasmid for each construct was transformed into Shuffle T7 Express cells (New England Biolabs) and plated on LB-agar plates with 150 µg/ml ampicillin at 30°C overnight. A single colony was picked and inoculated into 5 ml of LB with 150 µg/ml ampicillin and grown at 30°C overnight. The next day, the 5 ml culture was inoculated into 1 l LB with 150 µg/ml ampicillin and 0.2% glucose. The cells were cultured at 30°C, and 0.3 mM IPTG was added to induce protein expression once the OD_600_ reached 0.5. Protein expression was induced overnight at 16°C. The cells were harvested by centrifugation at 3500 rpm for 10 min using a Thermo Scientific ST40R centrifuge. The cell pellet from 2 l of culture was resuspended in 25 ml of column buffer (20 mM Tris-HCl, pH 7.4, 200 mM NaCl, 1 mM EDTA) and frozen by placing in the –80°C freezer until purification.

The frozen cells were thawed and lysed through 3 cycles of microfluidization at 20000 psi using a Microfluidizer Processor (Microfluidics). The cell lysate was centrifuged using an Eppendorf 5810R centrifuge at 12000 rpm for 30 min at 4°C to remove cell debris and insoluble proteins. 500 µl amylose resin was added to a column and was equilibrated with 5 ml of column buffer. The supernatant was added to the column and incubated on ice for 1 h on a rocking platform. Then the column was drained and washed with 5 ml column buffer twice. 5 ml of maltose elution buffer (20 mM Tris-HCl, pH 7.4, 200 mM NaCl, 1 mM EDTA, 20 mM maltose) was added to the column, and the column was incubated on ice for 30 min on a rocking platform. The eluted protein was collected in a 15 ml falcon tube, and then the column was eluted again with another 5 ml of maltose elution buffer. The eluted protein was concentrated to 4 ml using an Amicon ultra centrifugal filter (Millipore Sigma) and centrifuged using an Eppendorf 5425 centrifuge at 15000 rpm for 5 min at 4°C before being injected into an ӒKTA pure chromatography system (Cytiva). The protein was purified on a HiLoad 16/60 Superdex 200 prep-grade size exclusion chromatography column (Cytiva) connected to the FPLC system using an isocratic elution method with Tris gel filtration buffer (20 mM Tris-HCl, pH 7.4, 150 mM NaCl) as the mobile phase.

### Factor Xa protease reaction

The protein was concentrated to 1 mg/ml before the Factor Xa protease reaction. 20 µl protein was mixed with 1 µl of Factor Xa protease (New England Biolabs) in 100:1 *w*/*w* ratio. The reaction was incubated at room temperature for 3 h.

### Synthesis of (*E*)-2-methyl-2-butenoyl coenzyme A

MB-CoA (tiglyl-CoA) was synthesized using the method published in previous literature [52]. Tiglic acid (2.5 mg, 0.025 mmol), coenzyme A trilithium salt (10 mg, 0.0127 mmol), PyBOP (13 mg, 0.025 mmol), and potassium carbonate (7 mg, 0.05 mmol) were dissolved in 4 ml of THF/water (1:1). The reaction was incubated for 3 h at room temperature, and all the solvent was removed by rotary evaporator. The resulting white solid was purified by HPLC (Agilent, 1260 series) on a Phenomenex Luna 5μm C18 column 100 × 4.6 mm with a gradient of 5–95% methanol in water containing 5mM ammonium acetate over 30 min at a flow rate of 2 ml/min. HRMS(ESI) calculated for C_26_H_42_N_7_O_17_P_3_S [M+H]^+^ *m/z* 850.1644, found 850.1615. NMR and HRMS spectra are shown in Supplementary Figure S1-S2.

### Synthesis of (*E*)-2-methyl-2-butenoyl choline

2-(dimethylamino)ethyl (*E*)-2-methylbut-2-enoate: 1 ml of (*E*)-2-methylbut-2-enoyl chloride was added in 33 ml of diethyl ether and cooled down on ice. Then, 0.7 ml of 2-dimethylaminoethanol was added dropwise to the solution while stirring. The intermediate product was immediately observed as a white precipitate. Another 50 ml of diethyl ether was added to the reaction solution, and the solution was stirred at room temperature overnight. The reaction mixture was filtered through filter paper. The precipitated intermediate product was washed with diethyl ether and dissolved in 40 ml of 1M NaOH. The intermediate product was extracted from the aqueous solution using 80 ml of diethyl ether. The organic phase was separated and dried with MgSO_4_. The organic phase was filtered, and the solvent was removed using a rotary evaporator, yielding 0.43 g of a colorless liquid of the intermediate with a yield of 31.2%.

(*E*)-2-methylbut-2-enoyl choline: 0.43 g intermediate 2-(dimethylamino)ethyl (*E*)-2-methylbut-2-enoate was dissolved in 30 ml of diethyl ether and transferred to a three-necked round-bottom flask connected to a reflux condenser. 1 ml of iodomethane was added to the flask dropwise in the dark. The reaction was stirred for 12 h in the dark. The reaction mixture was filtered, and the precipitated product was air-dried. The product was purified by recrystallization using deionized water, yielding 0.25 g of yellow crystals with a yield of 53.4%. The total yield for the synthesis of (*E*)-2-methyl-2-butenoyl choline was 16.7%. ^1^H NMR (600 MHz, D_2_O) δ 6.92–6.86 (m, 1H), 4.57–4.53 (m, 2H), 3.75–3.71 (m, 2H), 3.19–3.16 (s, 9H), 1.77–1.75 (m, 6H); ^13^C NMR (150 MHz, D_2_O) 169.23, 141.05, 127.23, 64.82 (t, *J* = 0.02), 58.43, 53.91 (t, *J* = 0.03), 14.03, 11.32. HRMS(ESI) calculated for C_10_H_20_NO_2_ [M]^+^ *m/z* 186.1489, found 186.1493. NMR and HRMS spectra are shown in Supplementary Figure S4-S6.

### CEST-9.2 activity assay using synthetic substrates

MB-CoA, MB-carnitine (Sigma-Aldrich), MB-Choline were used for the assays. 50 mM MB-CoA, 20 mM MB-carnitine, and 10 mM MB-choline were initially dissolved in methanol, DMSO, and water, respectively, and then diluted to 1 mM in water before being added to the reactions. The reaction was set up with 100 µM MB substrates, 30 µM asc-ΔC9 (1 mg/ml stock dissolved in methanol) and 500 nM purified CEST-9.2 in either neutral buffer (50 mM Tris-HCl, pH 7.4, 100 mM NaCl) or acidic buffer (50 mM NaOAc, pH 5.2, 100 mM NaCl) in 50 µl total volume. The reactions were incubated at 25°C for 3 h or overnight. The reactions were quenched with 50 µl of LC-MS-grade methanol and centrifuged at 15000 rpm for 5 min before being analyzed using an Agilent 6130 quadrupole mass spectrometer.

Water with 0.1% formic acid was used as mobile phase A and acetonitrile with 0.1% formic acid was used as mobile phase B in the LC-MS method. A Phenomenex Luna 5 µm C18 column 100 × 4.6 mm was used for separating the analyte. The gradient elution was set up as follows: (1) 0–20 min: linearly increasing B from 5% to 60%; (2) 20–25 min: linearly increasing B from 60% to 100%; (3) 25–27 min: linearly decreasing B from 100% to 5%; (4) 27–30 min: holding at 5% B. The flow rate was maintained at 0.7 ml/min during a sample run. The [M + H]^+^, [M + Na]^+^, and [M-H]^-^ ions of the expected product, MB-asc-ΔC9, were monitored by both scan mode and SIM mode in both positive and negative mode.

### Cloning of yeast expression plasmids and yeast cell transformation

Each gene of interest was cloned into pPICZB plasmid or pPICZαA plasmid using the primers listed in Supplementary Table S2 and the restriction sites listed in Supplementary Table S1. 20 µg plasmid purified using miniprep kit (QIAGEN) was used for electroporation. The purified plasmid was digested using *Pme*I (New England Biolabs) for 4 h at 37°C. Briefly, 20 µg of DNA, 5 µl of *Pme*I, and 15 µl of 10X cutsmart buffer were added to a 150 µl reaction. 15 µl of ice-cold 3 M sodium acetate, pH 5.2, was mixed with the linearized DNA. Then, the DNA was mixed with 450 µl of ice-cold ethanol (200 proof) and was incubated on ice for 10 min. The precipitated DNA was centrifuged at 14000 rpm for 10 min at 4°C. The supernatant was removed, and the pellet was washed by being resuspended in 1 ml of 70% ethanol made from ethanol 200 proof at room temperature and being spun down at 14000 rpm for 10 min at 4°C. The supernatant was removed, and the pellet was dried by air flow for 5 min or until the edge of the pellet became transparent. The DNA pellet was then resuspended in 70 µl of DNase-free water.

The frozen GS200 competent cells were streaked on a YPD plate (1% yeast extract, 2% peptone, 2% dextrose, 2% agar) and incubated at 30°C overnight. A colony was inoculated into 5 ml of YPD (1% yeast extract, 2% peptone, 2% dextrose) starter culture and shaken at 30°C overnight. 500 µl of starter culture was inoculated into 500 ml of YPD in a 2.8 l baffled flask and was incubated for 12 h or until the OD_600_ was between 1.3 and 1.5. The cells were harvested by centrifugation at 3500 rpm for 5 min at 4°C. The supernatant was removed, and the cells were washed with 500 ml of ice-cold autoclaved water, then 250 ml of ice-cold autoclaved water, and then 20 ml of ice-cold 1M sorbitol. The cells were eventually resuspended in 1 ml of ice-cold 1M sorbitol and were kept on ice until electroporation. To maintain the sterility of the cells, the whole process was done on a Labconco horizontal clean bench.

80 µl of cells were mixed with 10 µg of linearized DNA and incubated on ice for 10 min. Then the mixture of cells and DNA was transferred to an ice-cold sterile, disposable electroporation cuvette (Fisher). Then the cells were electroporated using a MicroPulser Electroporator (Bio-Rad) on the mode specifically for *P. pastoris*. Immediately after the electroporation, the cells were mixed with 1 ml of ice-cold sorbitol by gently pipetting up and down to avoid cell death. The cells were transferred to a 15 ml falcon tube and incubated at 30°C without shaking for 1.5 h. Then 1 ml of YPD was added to the cells, and the cells were incubated and shaken at 250 rpm for 2 h at 30°C. After incubation, the cells were seeded onto a YPDS plate (1% yeast extract, 2% peptone, 2% dextrose, 1M sorbitol, 2% agar) supplemented with 100 µg/ml zeocin and incubated at 30°C in the dark. The colonies formed in about 2 or 3 d.

### CEST-9.2 expression and purification in yeast

The colonies on the YPDS plate supplemented with 100 µg/ml zeocin (1X) were restreaked on a YPD plate supplemented with 2 mg/ml zeocin (20X). The colonies that survived on the 20X zeocin plate were inoculated into 5 ml of BMGY (1% yeast extract, 2% peptone, 100mM potassium phosphate, pH 6.0, 1.34% YNB, 0.4 µg/ml biotin, 1% glycerol, 100 µg/ml zeocin) culture medium and shaken at 250 rpm for 20 h at 30°C. Then, the cells were spun down at 3500 rpm for 10 min, and the supernatant was removed. The cell pellet was resuspended in 5 ml of fresh BMGY and shaken at 250 rpm for another 20 h at 30°C. Then, the cells were spun down and resuspended in 5 ml BMMY (1% yeast extract, 2% peptone, 100 mM potassium phosphate, pH 6.0, 1.34% YNB, 0.4 µg/ml biotin, 0.5% methanol, 100 µg/ml zeocin) to induce the protein expression. After a 24-h shaking at 250 rpm at 30°C, 50 µl of methanol was added to the culture, and protein expression was induced for another 24 h. After 48 h of induction in total, the cells were spun down at 3500 rpm. The supernatant (conditioned culture medium) was isolated and concentrated to 200 µl using an Amicon ultra-centrifugal filter with a 10 kDa cutoff. The cell pellet was resuspended in 500 µl of Pichia lysis buffer A (50 mM sodium phosphate, pH 7.4, 5% glycerol, 1 mM EDTA, 1 mM PMSF). The resuspended cells were lysed by being vortexed at 3000 rpm for 10 min with glass beads (425-600 µm, Sigma-Aldrich) in 1:1 (*v*/*v*) ratio. The expression of protein was then detected by anti-His western blot.

For large-scale protein expression, the 5 ml BMGY starter culture was inoculated into 50 ml of BMGY medium and incubated at 30°C for 60 h. The 50 ml culture was then inoculated into 500 ml of BMGY medium and incubated at 30°C for 48 h. Then, the cells were spun down and resuspended in 500 ml of BMMY to induce protein expression. After a 24 h incubation at 30°C, 5 ml of methanol was added to the cell culture, and protein expression was induced for another 24 h at 30°C. After 48 h of induction in total, the cells were harvested and resuspended in 50 ml Pichia lysis buffer B (50 mM sodium phosphate, pH 7.4, 500 mM sodium chloride, 5% glycerol). The cells could not be frozen and stored for use later and instead had to be lysed on the same day of harvest by three cycles of microfluidization. The cell lysate was centrifuged using an Eppendorf 5810R centrifuge at 12000 rpm for 30 min at 4°C to remove the cell debris and insoluble proteins. 1 ml of Ni-NTA resin was added to a column and was equilibrated with 5 ml of Pichia lysis buffer B. The supernatant was added to the column and incubated on ice for 1 h on a rocking platform. Then, the column was drained and washed with 5 ml of Pichia lysis buffer B twice and 5 ml of Pichia washing buffer (50 mM sodium phosphate, pH 7.4, 500 mM sodium chloride, 5% glycerol, 20 mM imidazole) twice. 5 ml of Pichia elution buffer (50 mM sodium phosphate, pH 7.4, 500 mM sodium chloride, 5% glycerol, 250 mM imidazole) was added to the column, and the column was incubated on ice for 30 min on a rocking platform. The eluted protein was collected in a 15 ml falcon tube, and then the column was eluted again with another 5 ml of Pichia elution buffer. The eluted protein was concentrated to 4 ml using an Amicon ultra centrifugal filter (Millipore Sigma) and centrifuged using an Eppendorf 5425 centrifuge at 15000 rpm for 5 min at 4°C before FPLC purification. The FPLC purification was done in the same way as described above using Pichia gel filtration buffer (50 mM sodium phosphate, pH 7.4, 150 mM sodium chloride). The purified protein was detected by western blot using a 6xHis tag monoclonal antibody conjugated with DyLight 488 (Invitrogen). The uncropped western blot images are shown in Supplementary Figure S15.

### Generation and purification of bacmid for insect cell protein expression

Each gene of interest was cloned into pFastBac-1 plasmid using the primers listed in Supplementary Table S2 and the restriction sites listed in Supplementary Table S1. The frozen DH10Bac competent cells were thawed on ice for 5 min. Then the subcloned pFastBac-1 plasmids were added to the cells and incubated on ice for 20 min. The cells were heat-shocked at 42°C for 40 s. 900 µl of LB medium was added to the cells, and the cells were incubated at 37°C for 4 h with shaking at 150 rpm. The cells were seeded on an LB-agar plate supplemented with 50 µg/ml kanamycin, 7 µg/ml gentamycin, 10 µg/ml tetracycline, 100 µg/ml Bluo-gal, and 40 µg/ml IPTG. The plate was incubated at 37°C for 48 h in the dark. Ten white colonies were then restreaked on a new LB-agar plate supplemented with 50 µg/ml kanamycin, 7 µg/ml gentamycin, 10 µg/ml tetracycline, 100 µg/ml Bluo-gal, and 40 µg/ml IPTG and were incubated at 37°C overnight. The purely white colonies were inoculated into 10 ml of LB medium supplemented with 50 µg/ml kanamycin, 7 µg/ml gentamycin, and 10 µg/ml tetracycline and incubated at 37°C overnight. The cells were then harvested by centrifugation at 3500 rpm for 10 min. The bacmids were isolated from the cells using the PureLink HiPure miniprep kit (Thermo Fisher Scientific). The insertion of the gene of interest into the bacmid was verified by PCR using pUC/M13 forward primer (5′-CCCAGTCACGACGTTGTAAAACG-3′) and pUC/M13 reverse primer (5′-AGCGGATAACAATTTCACACAGG-3′) and Taq DNA polymerase (New England Biolabs). The PCR reaction was set up according to the manual of Taq DNA polymerase with the annealing temperature set to 52°C. The bacmids in which the gene of interest had been successfully integrated had a PCR product of 2300 bp plus the size of gene of interest, while the bacmids lacking the gene of interest had a PCR product of 300 bp.

### Transfection of Sf9 cells with the bacmid for generating baculovirus P1

The Sf9 insect cells were maintained in 30 ml Sf-900 II serum-free medium without any antibiotic-antimycotic selection in a 250 ml Erlenmeyer flask shaken at 150 rpm at 27°C before transfection. The density of the cells was checked every 24 h by counting the cells using a hemocytometer (Fisher Scientific). The cells were regularly passaged when the cell density was 2 × 10^6^ to 4 × 10^6^ cells ml^-1^ and inoculated at a cell density between 0.3 × 10^6^ to 0.5 × 10^6^ cells ml^-1^ to maintain the cells at a high viability. On the day of transfection, the cells were in log phase at a cell density of 1.5 × 10^6^ to 2.5 × 10^6^ cells ml^-1^. The cells were diluted in Grace’s medium (Gibco) to 0.4 × 10^6^ cells ml^-1^ in 15 ml total volume. 2 ml of diluted cells were added to each well of a tissue culture-treated six-well plate (Fisher Scientific). The cells were incubated at 27°C for 30 min or until the cells settled to the bottom of the wells. 8 µl of Cellfectin II reagent (Thermo Fisher Scientific) was mixed with 100 µl of Grace’s medium, and 1 µg of bacmid was mixed with 100 µl of Grace’s medium. The two mixtures were combined, mixed gently, and incubated at room temperature for 15 to 30 min. Then the mixture was added to each well dropwise. The plate was incubated at 27°C for 3 to 5 h. Then the medium was removed and replaced with 2 ml of Grace’s medium supplemented with 10% fetal bovine serum (FBS). The transfected cells were incubated at 27°C for 72 h. After 72 h, cells became detached, which is the sign of virus infection. When collecting baculovirus P1, the cells were resuspended by pipetting up and down. Then, the mixture of cells and medium was centrifuged at 500 g for 5 min at 4°C to spin down the cell debris. Then the clarified baculovirus P1 was transferred to a sterile 2 ml microcentrifuge tube and stored at 4°C for months.

### Viral plaque assay for determining the baculovirus titer

On the day of performing the viral plaque assay, 30 ml of cells grown in 250 ml Erlenmeyer flask were in log phase at a cell density between 1.5 × 10^6^ and 2.5 × 10^6^ cells ml^-1^. Around 3 ml to 5 ml cells were diluted to 15 ml at a density of 0.5 × 10^6^ cells ml^-1^ using Sf-900 II serum-free medium. 2 mL of cells were added to each well and incubated at 27°C for 30 min or until the cells settled to the bottom of the wells and became attached. A serial dilution of the baculovirus at 1:10^-2^, 1:10^-3^, 1:10^-4^, 1:10^-5^, 1:10^-6^, and 1:10^-7^ in 1 ml of Sf-900 II serum-free medium was prepared. The medium was removed from the wells after the cells were attached to the bottom of the wells. 500 µl of diluted baculovirus was added to each well and incubated at 27°C for 1 to 1.5 h. Fresh plaque medium was prepared by mixing 10.25 ml of Sf-900 II 1.3X medium, 0.825 ml of heat-inactivated FBS, and 82.5 µl of 100X antibiotics-antimycotics (Thermo Fisher Scientific) with 5 ml of 4% agarose (Fisher Scientific) dissolved in water and sterilized by autoclave. All reagents for preparing plaque medium were incubated at 60°C for 15 min before the mixing to avoid the solidification of agarose. The baculovirus was removed from the wells, and then, 2 ml of plaque medium was added to each well. After the agarose solidified, 2 ml of Sf-900 serum-free medium was added to each well. The plate was incubated at 27°C for 4 d. The plaque staining buffer was prepared by mixing 0.33% Neutral Red solution (Sigma-Aldrich) and 1X PBS buffer, pH 7.4 (Gibco) in 1:11 ratio on the day of staining the plaques. The medium was removed and replaced with 2 ml of plaque staining buffer. After 2 h of staining, the plaque staining buffer was removed, and the plate was incubated upside down at 27°C in the dark. The number of plaques formed was counted the next day.

### Protein expression using Sf9 cells in a 24-well plate

On the day of infecting the Sf9 cells for protein expression, the cells were in log phase at a cell density between 1.5 × 10^6^ and 2.5 × 10^6^ cells ml^-1^ and diluted to 0.6 × 10^6^ cells ml^-1^. 1 ml of cells were seeded in each well of a tissue culture-treated 24-well plate and incubated at 27°C for 30 min or until the cells settled to the bottom of the wells. The medium was removed and replaced with 300 µl of fresh Sf-900 serum-free medium. The baculovirus was added to each well at a desired multiplicity of infection (MOI) (e.g., 2, 5, 10, 20). The volume of baculovirus required for a specific MOI equals the number of cells times the MOI divided by the viral titer.

The infected cells were incubated at 27°C for various times. For membrane proteins (e.g., full-length CEST-9.2), protein expression was detected by western blot 72 to 96 h after infection. For secreted proteins, protein expression was detected by western blot 48 to 72 h after infection. To generate the samples for western blot, the cells were firstly resuspended in the culture medium by pipetting up and down and then spun down at 500 g for 5 min. The condition medium (supernatant) was transferred to a separate tube and mixed with 2 × Laemmli sample buffer (Bio-Rad). The cells were lysed up in 50 µl of Sf9 SDS lysis buffer (62.5 mM Tris, pH 6.8, 2% SDS) by being vortexed for 5 min. Then, 50 µl of 2 × Laemmli sample buffer was mixed with the cell lysate. 20 µl of sample was loaded to each well in the SDS-PAGE gel. The protein was detected by western blot using 6xHis tag monoclonal antibody conjugated with DyLight 488 (Invitrogen) and StrepTactin-HRP (IBA Lifesciences) for anti-His and anti-Strep western blot, respectively. The uncropped western blot images are shown in Supplementary Figure S15.

### Purification of secreted CEST-9.2 from Sf9 cell culture medium

500 ml of cells in the log phase at a cell density between 1.5 × 10^6^ and 2.5 × 10^6^ cells mL^-1^ were infected by adding baculovirus to the cell culture with an MOI of 2. The cells were grown for another 48 h, and the culture medium containing secreted CEST-9.2 was isolated by centrifugation at 3500 rpm for 10 min. The supernatant was then transferred to a clean Erlenmeyer flask. For the purification of His-tagged CEST-9.2, 1 ml of Ni-NTA resin was added to the medium, and the medium was stirred at 4°C for 1 h. Then, the medium was loaded onto a column, and the column was drained by gravity. The column was washed by 10 ml of wash buffer (50 mM HEPES, pH 7.4, 500 mM NaCl) twice. Then, 5 ml of elution buffer (50 mM HEPES, pH 7.4, 500 mM NaCl, 250 mM imidazole) was added to the column, and the column was incubated on ice for 30 min on a rocking platform. The eluted protein was collected in a 15 ml centrifuge tube, and then the column was eluted again with another 5 ml of elution buffer. The eluted protein was concentrated to 4 ml using an Amicon ultra centrifugal filter (Millipore Sigma) and centrifuged using an Eppendorf 5425 centrifuge at 15000 rpm for 5 min at 4°C before FPLC purification. The FPLC purification was done in the same way described above using HEPES gel filtration buffer (25 mM HEPES, pH 7.4, 150 mM NaCl). For the purification of strep-tagged CEST-9.2, StrepTactinXT 4Flow resin (IBA Lifesciences) was used for packing the column, buffer W (100mM Tris-HCL pH7.5, 150mM NaCl) was used for washing the column, buffer BXT (100mM Tris-HCL pH7.5, 150mM NaCl, 50mM biotin) was used for eluting the protein, and TBS Buffer (25mM Tris-HCl, pH 7.5, 150mM NaCl) was used for FPLC.

### Hydrolysis assay using *p*-nitrophenyl acetate

The hydrolysis reaction was set up with 200 µM of *p*-nitrophenyl acetate and 500 nM of CEST-9.2 in either neutral buffer (50 mM Tris-HCl, pH 7.4, 100 mM NaCl) or acidic buffer (50 mM NaOAc, pH 5.2, 100 mM NaCl) in 150 µl total volume. The production of *p*-nitrophenol (320 nm) or *p*-nitrophenoxide (400 nm) at pH 5.2 and pH 7.4, respectively, was monitored using a UV-vis spectrometer at 1s intervals. The initial rates of the reaction were calculated using the extinction coefficients for *p*-nitrophenol (4300 M^-1^cm^-1^ at 320 nm) or *p*-nitrophenoxide (18300 M^-1^cm^-1^ at 400 nm). For blank controls, uninfected Sf9 culture medium instead of the enzyme was added to the reaction.

### Expression, purification, and protease reaction of Super TEV II protease

Rosetta (DE3) cells transformed with Super TEV II protease-expressing plasmid [69] were cultured in 2 l of LB medium supplemented with 150 µg/ml ampicillin at 37°C until the OD_600_ reached 0.7. 1 mM of IPTG was added to the culture, and protein expression was induced at 18°C overnight. The cells were harvested at 3500 rpm for 10 min and then resuspended in 25 ml of TEV lysis buffer (20 mM HEPES, pH 7.5, 500 mM NaCl, 10% glycerol). The resuspended cells were lysed by three cycles of microfluidization, and the lysate was immediately centrifuged at 12000 rpm for 30 min at 4°C to pellet cell debris and insoluble proteins. Then, the supernatant was loaded into a column with 1 ml of Ni-NTA resin equilibrated with 5 ml TEV lysis buffer. The column was incubated on ice for 1 h on a rocking platform. The column was drained by gravity and washed with 5 ml of TEV lysis buffer twice and 5 ml of TEV wash buffer (20 mM HEPES, pH 7.5, 500 mM NaCl, 10% glycerol, 20 mM imidazole) twice. The column was incubated with 5 ml of TEV elution buffer (20 mM HEPES, pH 7.5, 500 mM NaCl, 250 mM imidazole) for 30 min on ice, and the eluted protein was collected in a 15 ml centrifuge tube. The column was eluted with another 5 ml of TEV elution buffer, and the two elution fractions were combined, added to a SnakeSkin Dialysis Tubing with 10 kDa cutoff (Thermo Fisher Scientific), and dialyzed in 1 l of TEV dialysis buffer (20 mM HEPES, pH 7.5, 100 mM NaCl) at 4°C overnight. The dialyzed protein was concentrated to 4 mg/ml using an Amicon ultra centrifugal filter (Millipore Sigma) and mixed with glycerol in a 1:1 ratio. The aliquoted Super TEV II protease in 500 µl each was flash frozen in liquid nitrogen and then stored at −80°C for later use.

The frozen Super TEV protease II was thawed at room temperature before use. The Super TEV II protease and 0.5 mg substrate protein were mixed in a 1:50 (*w*/*w*) ratio in TEV dialysis buffer. The reaction was incubated at 30°C for 1 h. Then the reaction product was diluted to 5 ml, and the TEV protease was removed by Ni-NTA resin on a gravity column. The column flow-through fraction containing tag-free CEST-9.2 was collected and used for downstream enzymatic assays.

### Circular dichroism spectroscopy of CEST-9.2

CEST-9.2 was diluted to 0.1 mg/ml in TBS buffer. The CD spectrum of CEST-9.2 from 200 nm to 280 nm was measured by Chirascan V100 (Applied Photophysics, Inc.). A blank spectrum of TBS buffer was used for background subtraction.

### Dynamic light scattering of CEST-9.2

The protein was concentrated to 1.5 mg/ml. 1 ml of concentrated protein was added to a disposable cuvette and measured by Malvern Zetasizer Nano ZS using protein size mode.

### LC-MS analysis of candidate substrates in wild-type and *cest-9.2* mutant worms

2,000,000 synchronized wild-type (N2) or *cest-9.2* L1 larvae were cultured in 25 ml of S medium shaken at 225 rpm at 22.5°C and fed with 5 ml of 25X OP50 bacteria every 24 h. The worms were harvested at 800 g for 5 min when the worms were young adults. The worm pellet was frozen at −80°C overnight and then lyophilized to dryness. The dried sample was ground into a fine powder using a mortar and pestle and extracted with 15 ml of ethanol (200 proof) at room temperature for 3 h on a shaker at 150 rpm. The dissolved sample was filtered through cotton in a long Pasteur pipette. The sample was concentrated to 2 ml using a rotary evaporator. The concentrated sample was filtered through cotton in a long Pasteur pipette and dried completely using a SpeedVac vacuum concentrator. The extracted sample was resuspended in 150 µl of 50% ethanol and centrifuged at 15000 rpm for 5 min. Then, the supernatant was analyzed by LC-MS using the same method described previously. Synthetic standards for MB-CoA, MB-carnitine, and MB-choline were injected into LC-MS as references with retention times of 7.8 min, 6.3 min, and 5.8 min, respectively [70].

## Supplementary material

online supplementary material 1.

## Data Availability

The data presented in this study are available within the main article and its supplementary file. The computational models are available on GitHub (https://github.com/HelloWorld2031/CEST-9.2_AutoDock_Model.git). [67]

## Abbreviations

AChE: acetylcholinesterase
BChE: butyrylcholinesterase
CD: circular dichroism
CEST: carboxylesterase domaincontaining
CESs: carboxylesterases
CoA: coenzyme A
ER: endoplasmic reticulum
IC: indole-3-carbonyl
LROs: lysosome-related organelles
MB: (*E*)-2-methyl-2-butenoyl
MBP: maltose binding protein
OS: octopamine succinyl
PABA: *para*-aminobenzoic acid
UGL: uric acid gluconucleoside
