# Supplementary material for: Expression, purification, and in vitro characterization of the carboxylesterase CEST-9.2 from Caenorhabditis elegans
Source: Biochem J. 2026 Jan 21;46(Pt 1):BSR20253840. doi: 10.1042/BSR20253840 (PMC12931606; doi:10.1042/BSR20253840)
Supplement: online supplementary material 1. [file bcj-46-1-BSR20253840-s001.pdf]

Supplementary Information

Expression, purification, and *in vitro* characterization of the carboxylesterase CEST-9.2 from *Caenorhabditis elegans*

Weijie Xu, Subhradeep Bhar, Steven D. Bruner, and Rebecca A. Butcher*

Department of Chemistry, University of Florida, Gainesville, FL 32611

*Correspondence: butcher@chem.ufl.edu

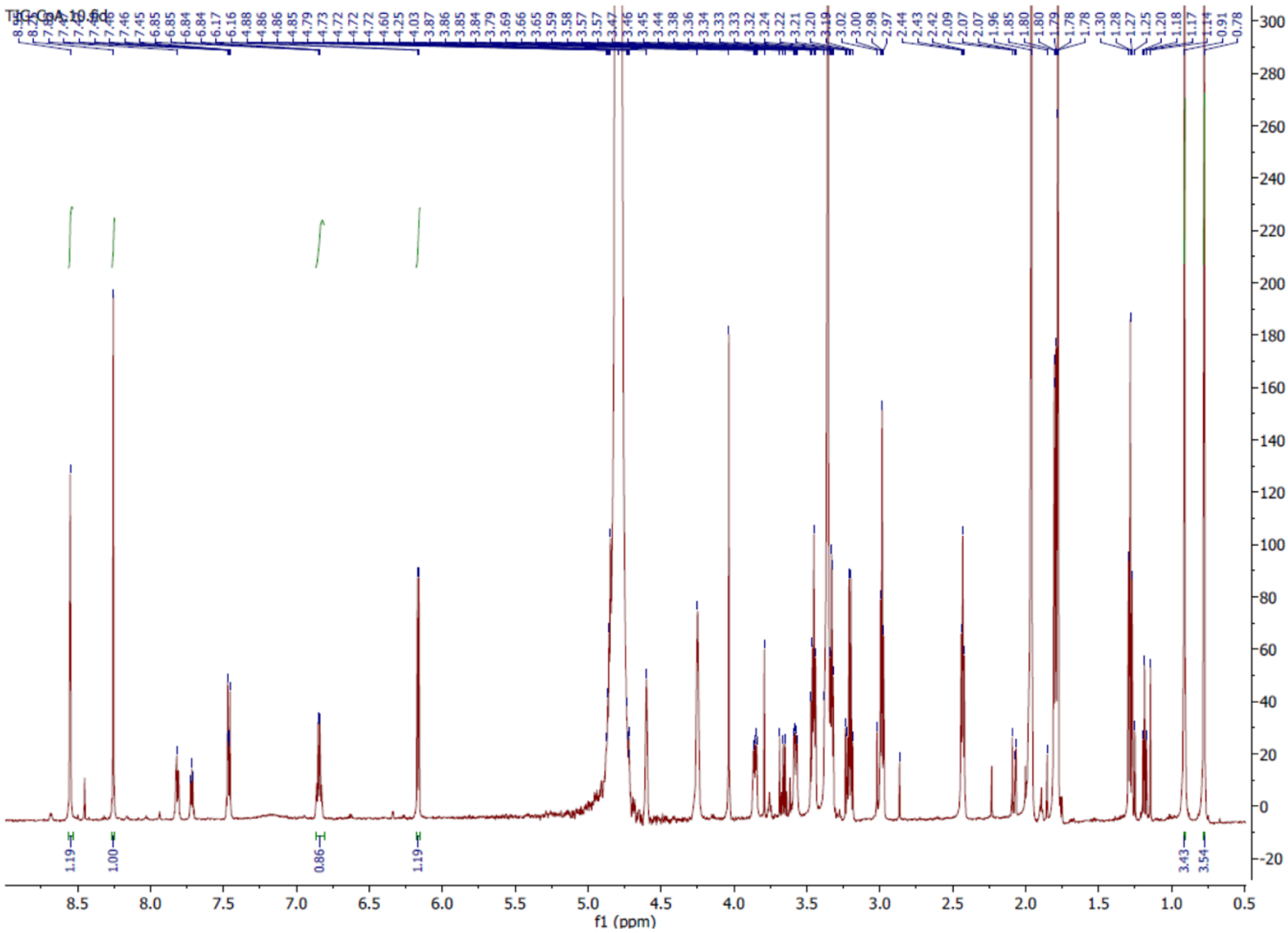

Figure S1. The ^{1}H-NMR spectrum of MB-CoA.

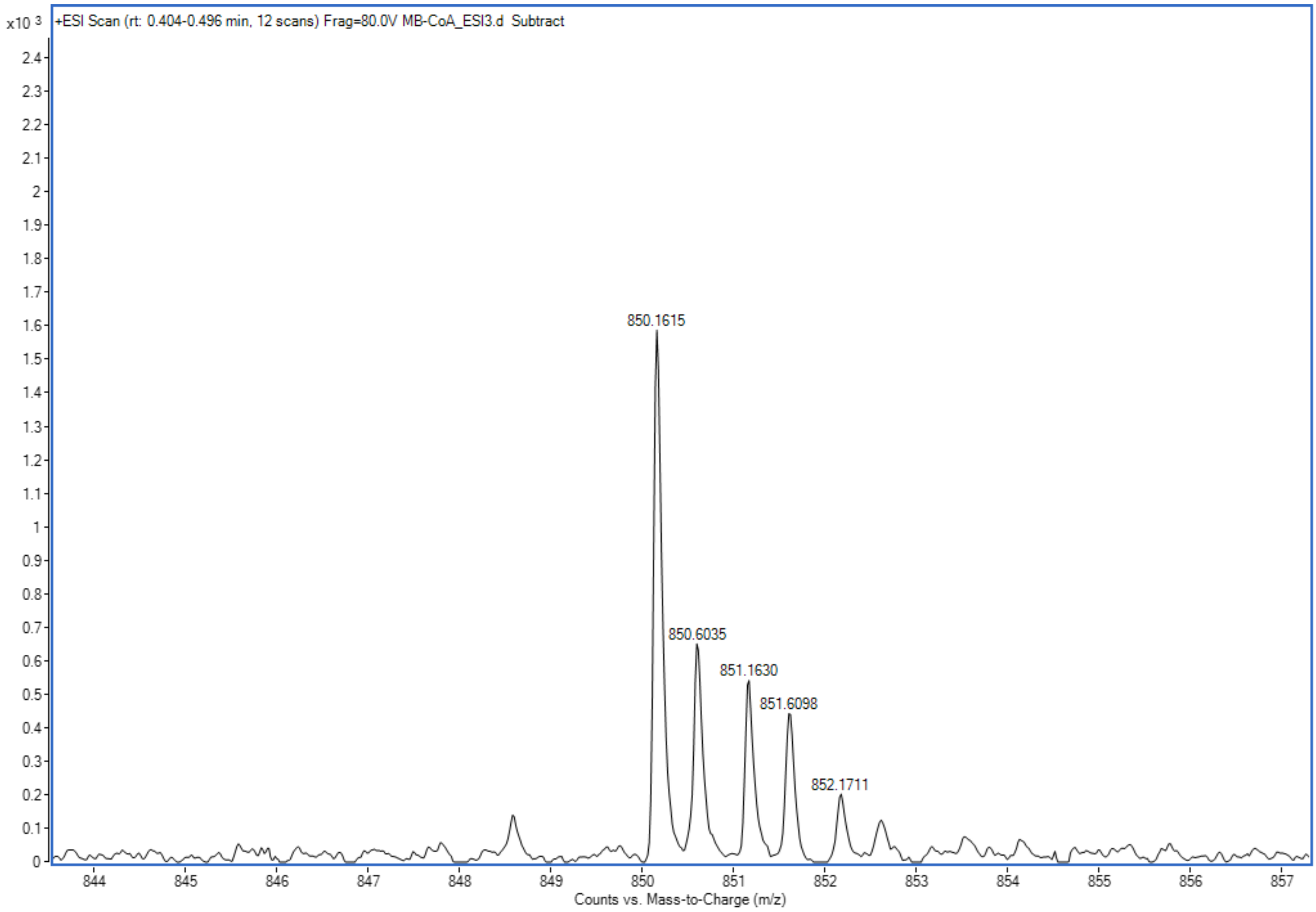

Figure S2. The HRMS spectrum of MB-CoA.

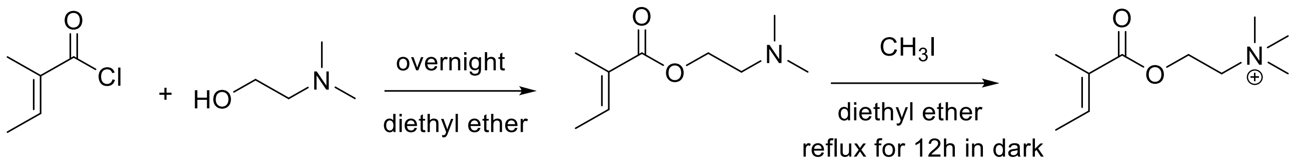

Figure S3. The scheme of the synthesis of MB-choline.

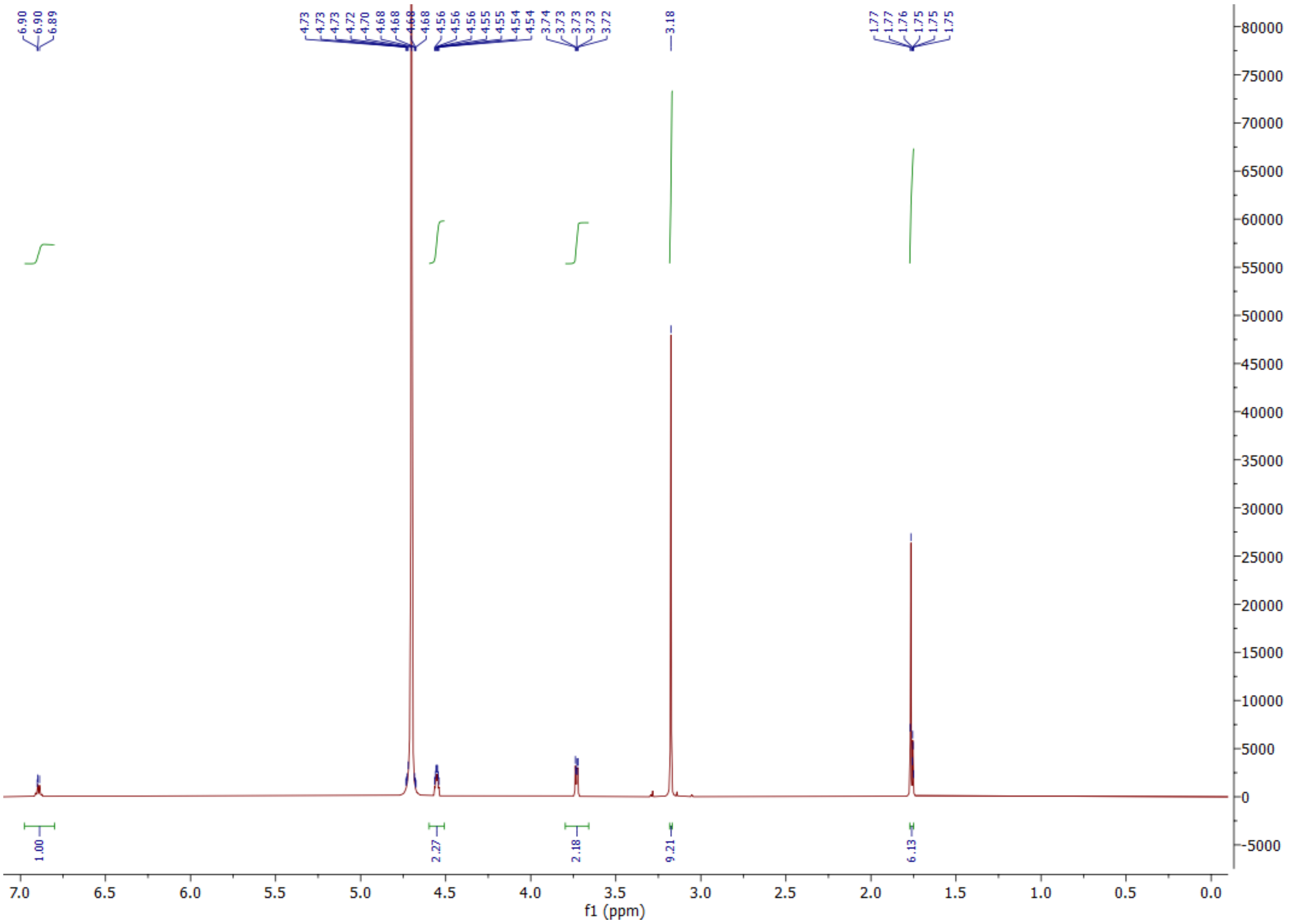

Figure S4. The ^{1}H-NMR spectrum of MB-choline.

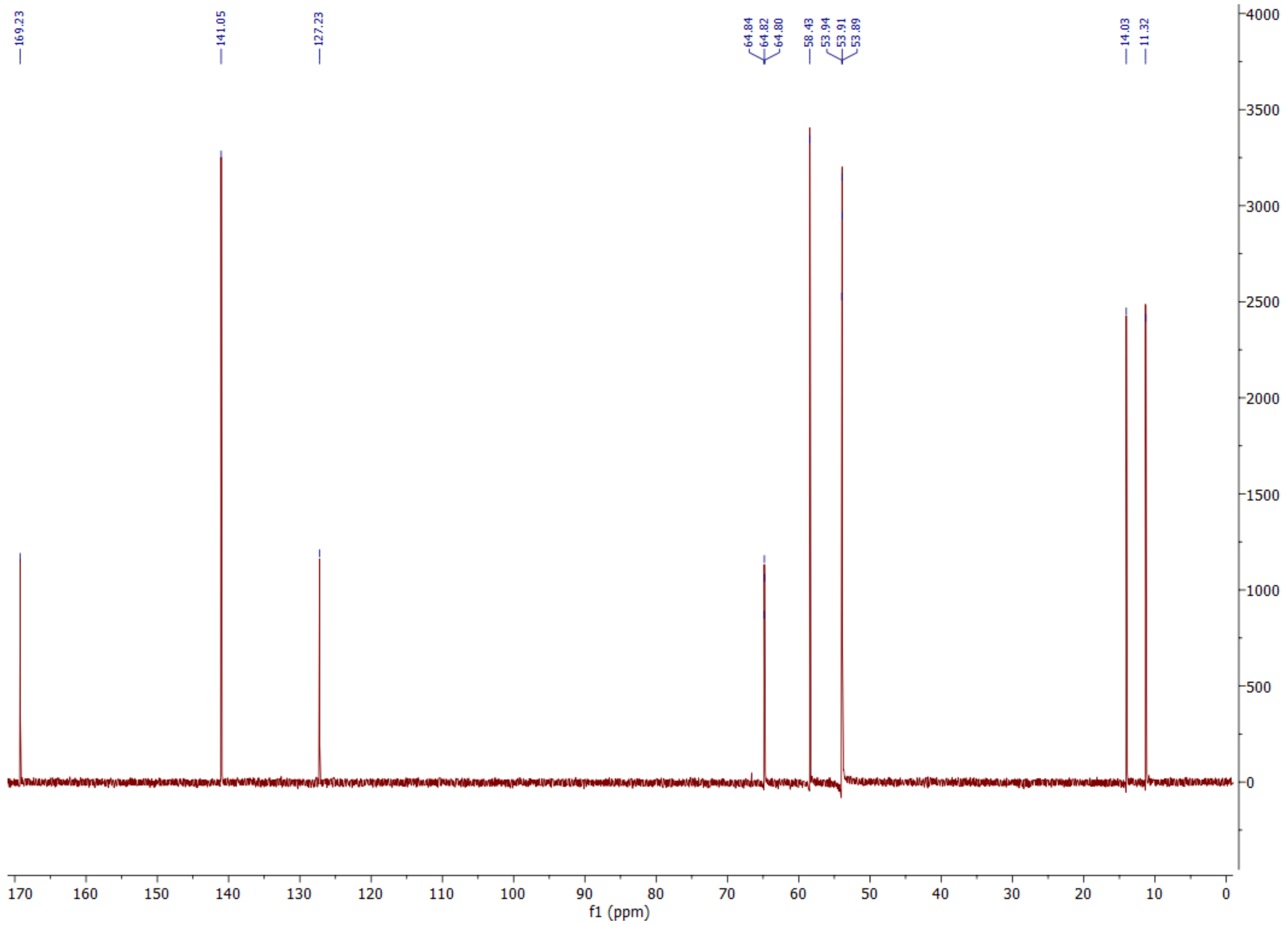

Figure S5. The ^{13}C-NMR spectrum of MB-choline.

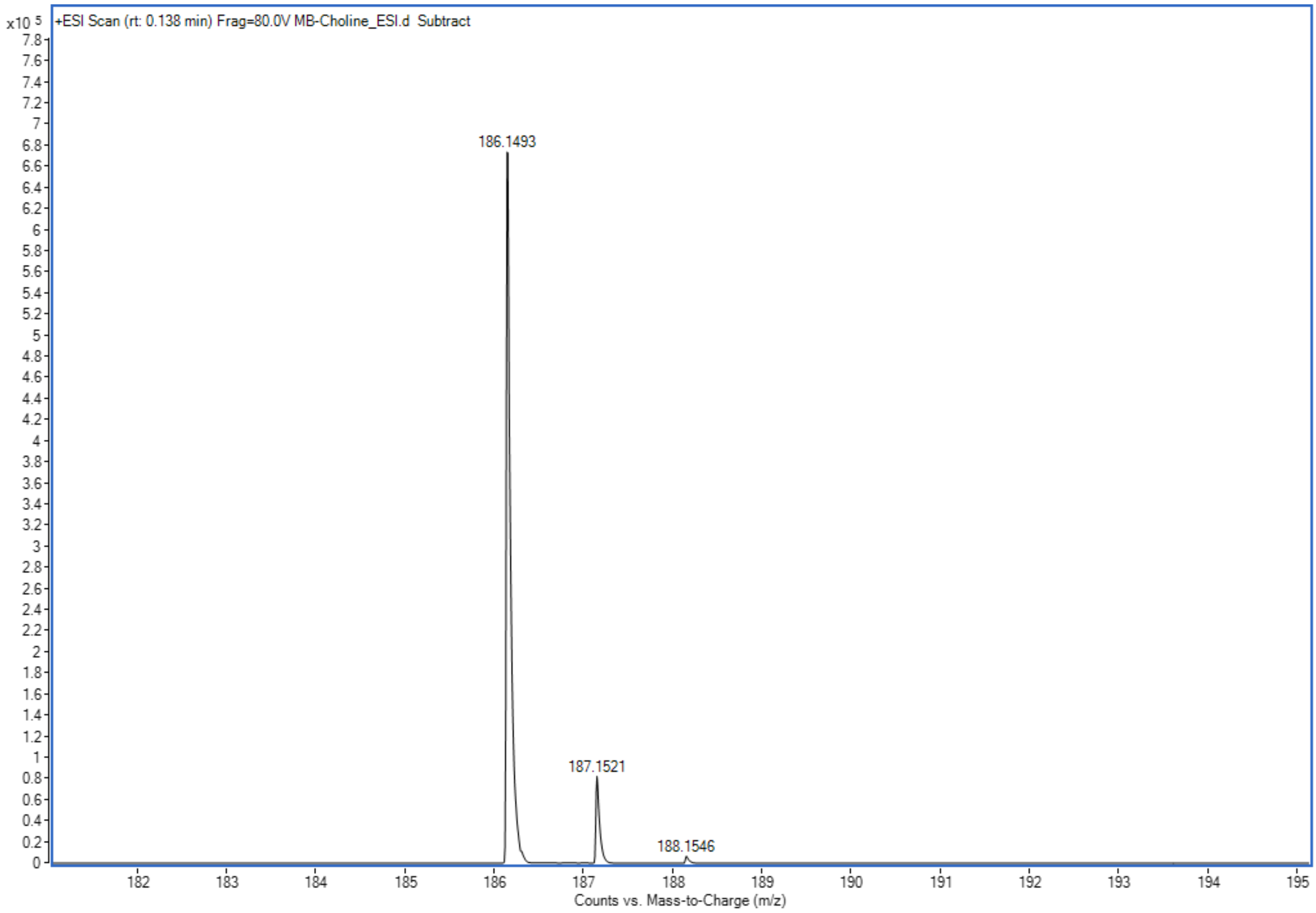

Figure S6. The HRMS spectrum of MB-choline.

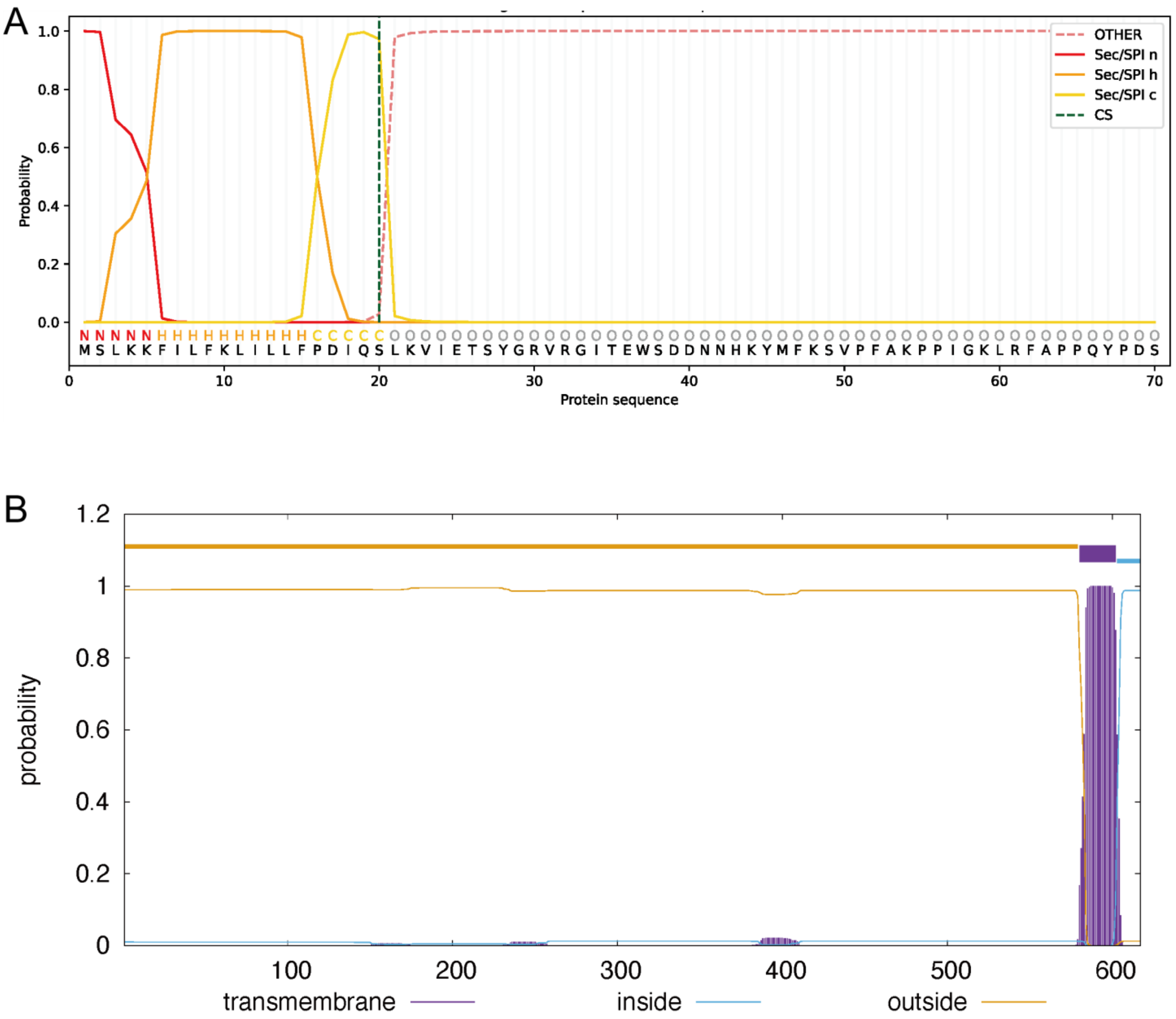

Figure S7. Sequence analysis of CEST-9.2. (A) The N-terminal signal peptide predicted by Signal P 6.0. (B) The transmembrane domain of CEST-9.2 predicted by TMHMM 2.0.

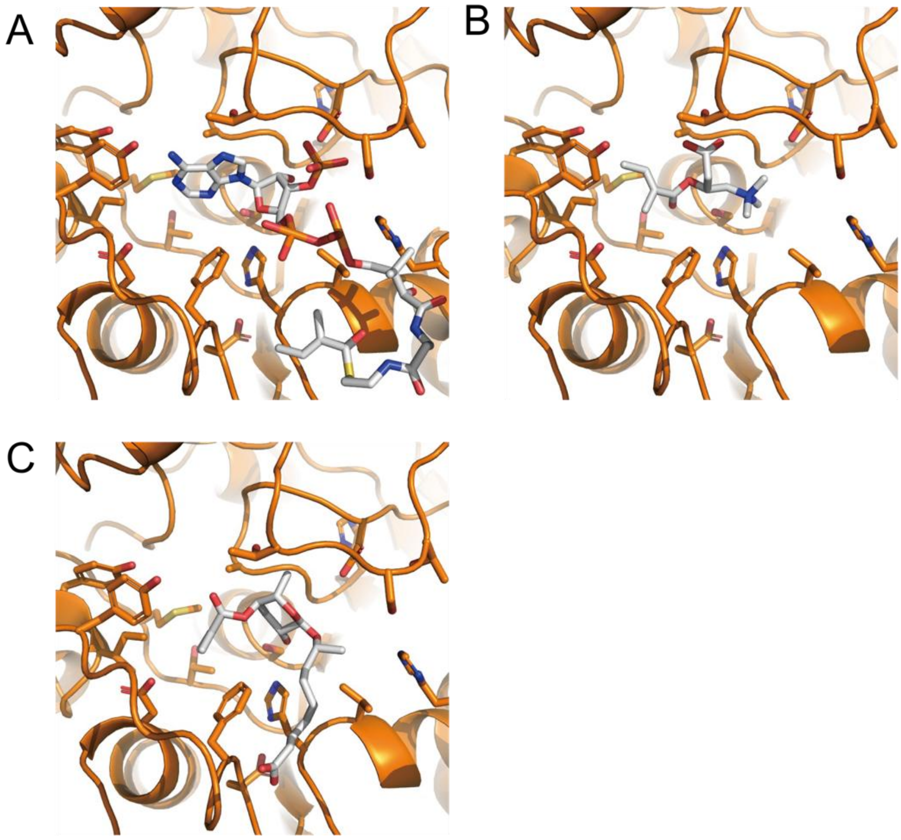

Figure S8. *In silico* interactions between CEST-9.2 and its proposed ligands. The proposed substrates and product, (A) MB-CoA, (B) MB-carnitine and (C) MB-asc-ΔC9, were docked in the AlphaFold model of CEST-9.2 using AutoDock vina 1.2.5.

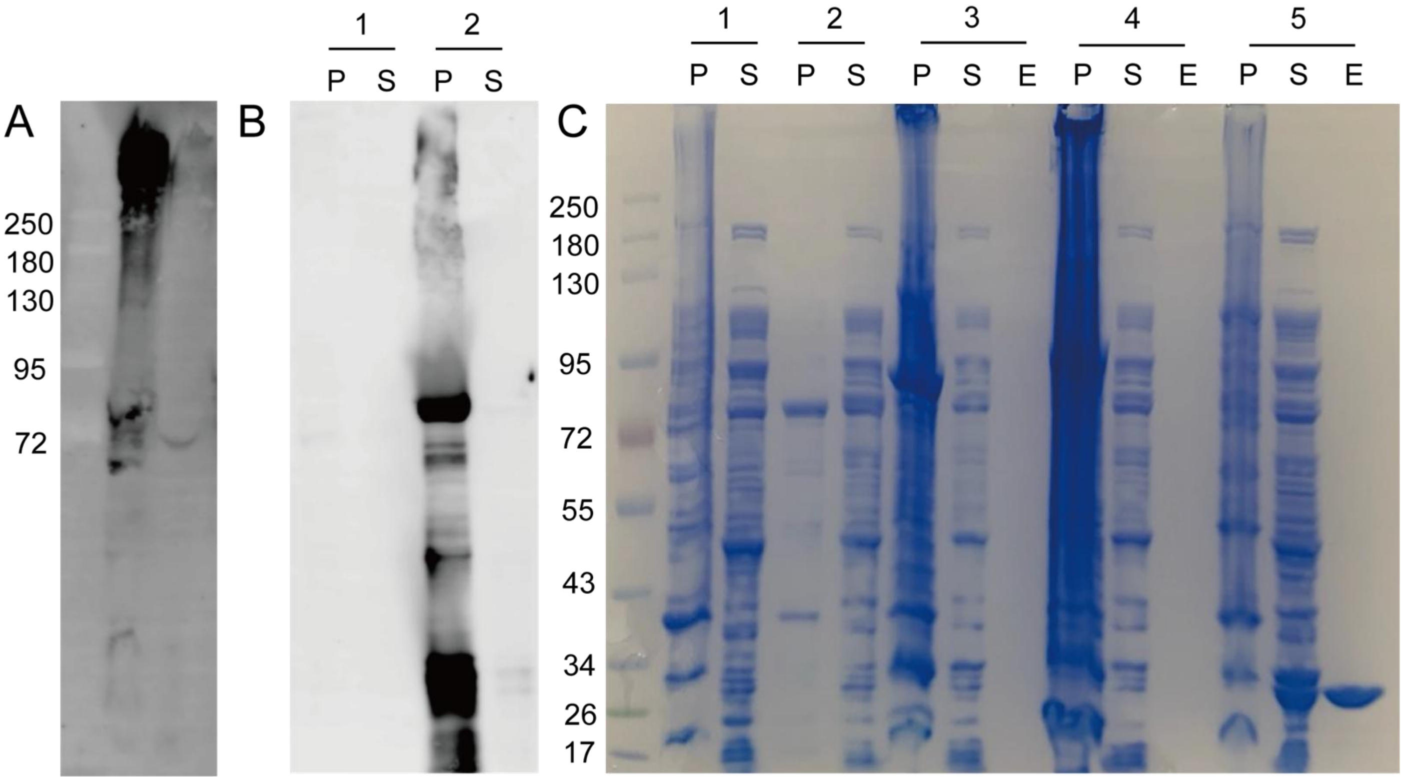

Figure S9. Full-length CEST-9.2 expression in *E. coli*. (A) Full-length CEST-9.2 (Ecoli#1) was expressed in insoluble fraction in C41(DE3) cells. (B) Full-length CEST-9.2 (Ecoli#1) was expressed in insoluble fraction in SHuffle T7 Express cells. (C) GST-tagged truncated CEST-9.2 enzymes (Ecoli#2 and Ecoli#3) were expressed in insoluble fractions in SHuffle T7 Express cells (P: cell pellet, S: supernatant, 1: pET-28-a empty vector, 2: Ecoli#1, 3: Ecoli#2, 4: Ecoli#3, 5: pGEX-6p-2 empty vector).

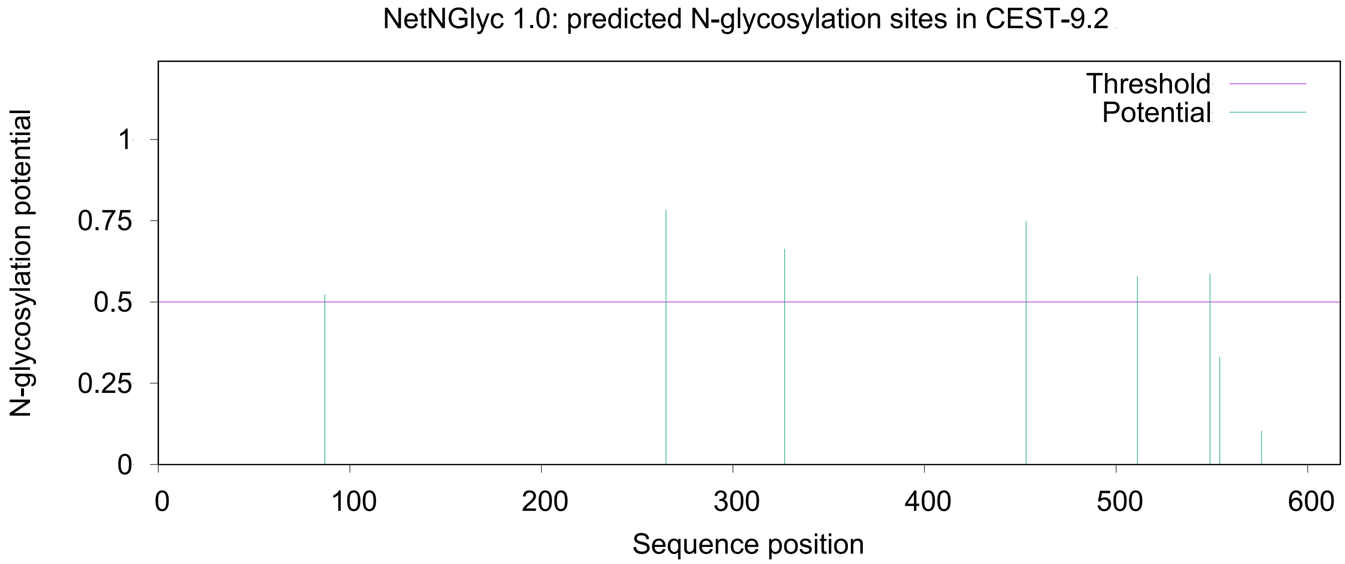

Figure S10. The predicted *N*-glycosylation sites in CEST-9.2. N265, N327, N453, N549 and N576 are predicted to be glycosylated using NetNGlyc 1.0 server.

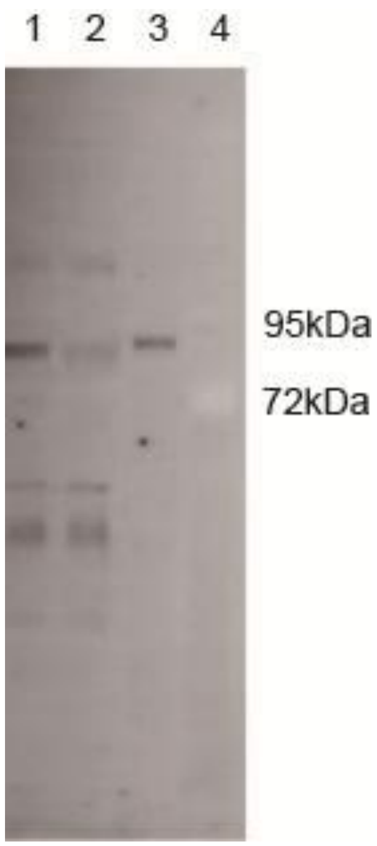

Figure S11. The glycosylation analysis of pPICZ#5. 1: protein treated with endo H_f at 37°C for 1h; 2: protein treated with endo H_f at 37°C overnight; 3: untreated protein; 4: marker.

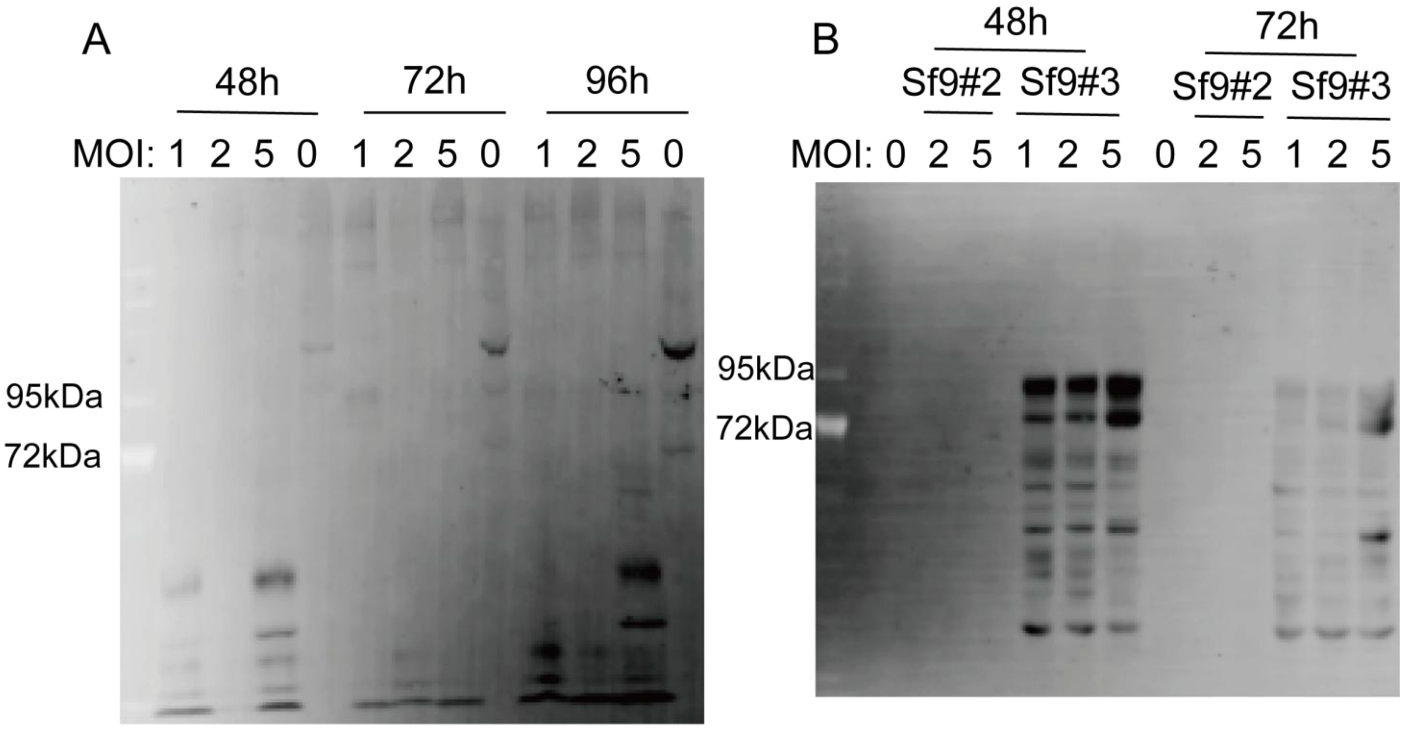

Figure S12. The expression of Sf9#1, Sf9#2 and Sf9#3. (A) The full-length Sf9#1 was expressed intracellularly but cleaved near C-terminus. (B) The longer truncated version of CEST-9.2 (Sf9#3) was expressed intracellularly in Sf9 cells.

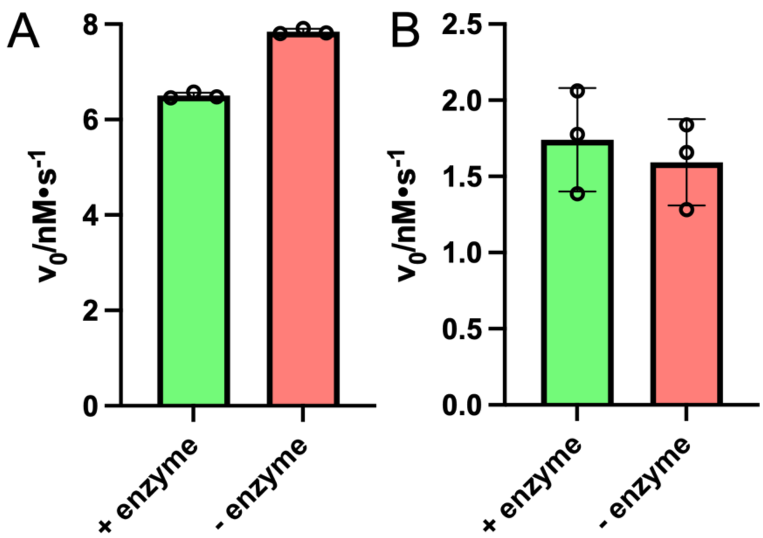

Figure S13. The *p*-nitrophenyl acetate hydrolysis assay using CEST-9.2 expressed in Sf9 cells. The initial rates of the hydrolysis reactions at (A) pH 7.4 or (B) pH 5.2 were measured within 30s of initiating the reactions. Data represent the mean of n = 3 replicates ± standard deviation.

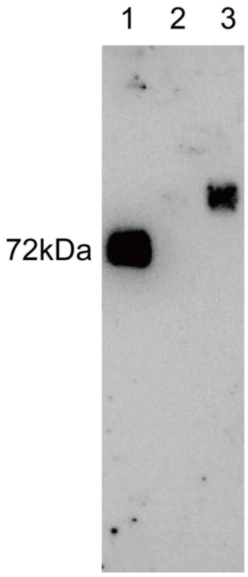

Figure S14. Removal of strep tag II from CEST-9.2 expressed using Sf9#14. 1. Standard protein ladder; 2. Sf9#14 treated with Super TEV protease at 30°C for 1h; 3. Sf9#14 incubated at 30°C for 1h (no protease).

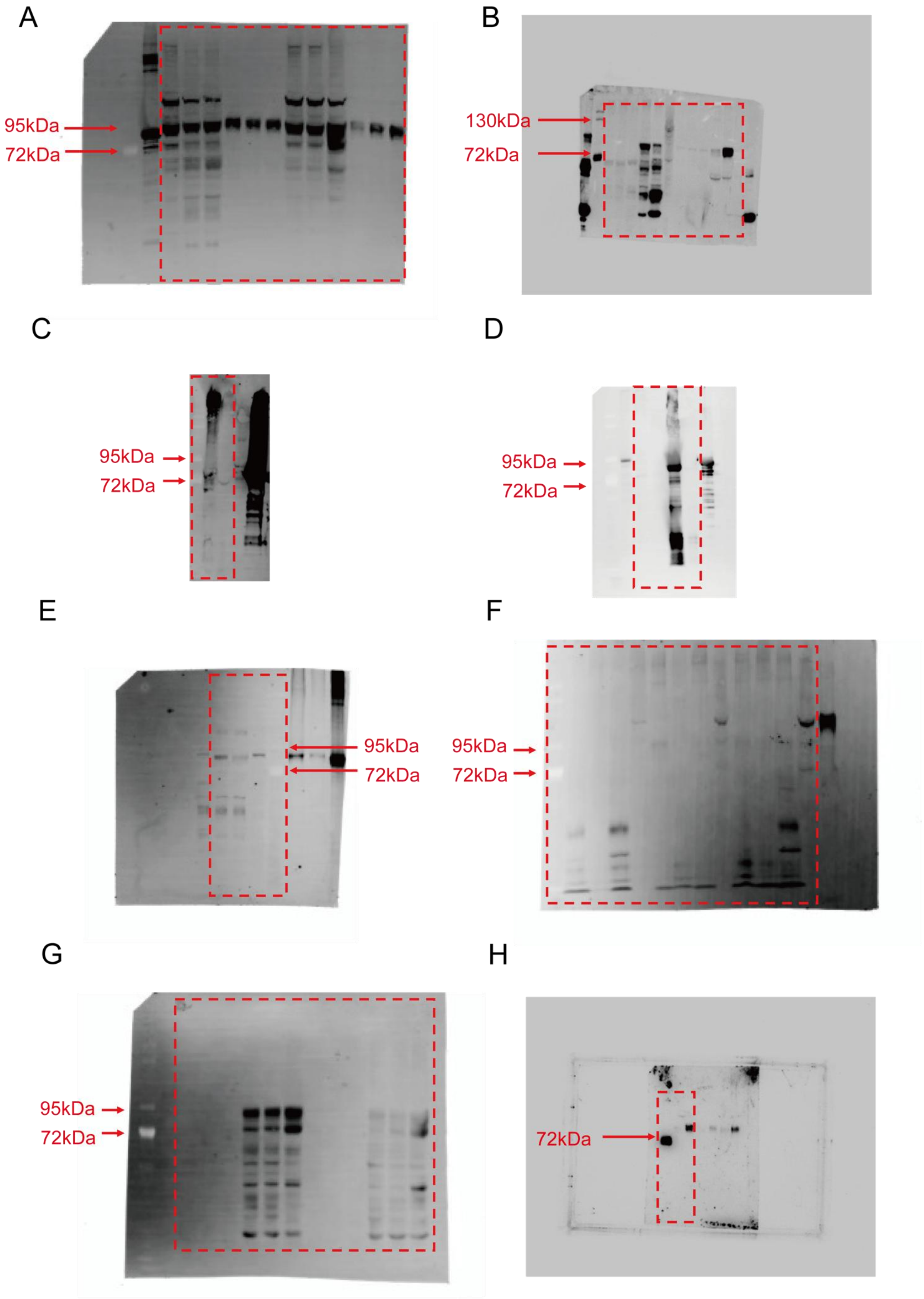

Figure S15. The uncropped western blot images. The cropped areas highlighted with red boxes are used in (A) Figure 6B, (B) Figure 6C, (C) Figure S9A, (D) Figure S9B, (E) Figure S11, (F) Figure S12A, (G) Figure S12B, and (H) Figure S14.

Table S1. The constructs made for CEST-9.2 expression.

Construct	Vector	Restriction Sites	Primers [a]	Sequence	Tag
Ecoli#1	pET-28-a	*BamH*I, *Xho*I	1, 2	1-617	N-terminal 6xHis tag
Ecoli#2	pGEX-6p-2	*BamH*I, *Xho*I	1, 3	1-539	N-terminal GST tag
Ecoli#3	pGEX-6p-2	*BamH*I, *Xho*I	1, 4	1-579	N-terminal GST tag
Ecoli#4	pMAL-c5x	*BamH*I, *Hind*III	1, 5	1-539	N-terminal MBP tag
Ecoli#5	pMAL-c5x	*BamH*I, *Hind*III	1, 6	1-559	N-terminal MBP tag
Ecoli#6	pMAL-c5x	*BamH*I, *Hind*III	1, 7	1-579	N-terminal MBP tag
Pichia#1	pPICZB	*Xho*I, *Xba*I	8, 10	1-617	C-terminal 6xHis tag
Pichia#2	pPICZB	*Xho*I, *Xba*I	8, 11	1-539	C-terminal 6xHis tag
Pichia#3	pPICZB	*Xho*I, *Xba*I	8, 12	1-579	C-terminal 6xHis tag
Pichia#4	pPICZαA	*Xho*I, *Xba*I	9, 11	21-539	N-terminal α-factor & C-terminal 6xHis tag
Pichia#5	pPICZαA	*Xho*I, *Xba*I	9, 12	21-579	N-terminal α-factor & C-terminal 6xHis tag
Pichia#6	pPICZαA	*EcoR*I, *Xba*I	13, 16	21-579	N-terminal α-factor & internal 6xHis tag
Pichia#7	pPICZαA	*Xho*I, *Xba*I	14, 10	21-617	N-terminal α-factor C-terminal 6xHis tag
Pichia#8	pPICZαA	*Xho*I, *Xba*I	15, 17	21-617	N-terminal α-factor & internal 6xHis tag
Sf9#1	pFastBac-1	*BamH*I, *Xho*I	18, 19	1-617	C-terminal 6xHis tag
Sf9#2	pFastBac-1	*BamH*I, *Xho*I	18, 20	1-539	C-terminal 6xHis tag
Sf9#3	pFastBac-1	*BamH*I, *Xho*I	18, 21	1-579	C-terminal 6xHis tag
Sf9#4	pFastBac-1	*BamH*I, *Xho*I	22, 2: 23, 2: 24, 2	21-617	Native signal peptide & N-terminal 10xHis tag and TEV site
Sf9#5	pFastBac-1	*BamH*I, *Xho*I	22, 2: 25, 2: 26, 2	21-617	Melittin signal peptide & N-terminal 10xHis tag and TEV site
Sf9#6	pFastBac-1	*BamH*I, *Xho*I	22, 2: 27, 2: 28, 2	21-617	gp64 signal peptide & N-terminal 10xHis tag and TEV site
Sf9#7	pFastBac-1	*BamH*I, *Xho*I	22, 4: 23, 4: 24, 4	21-579	Native signal peptide & N-terminal 10xHis tag and TEV site
Sf9#8	pFastBac-1	*BamH*I, *Xho*I	18, 29; 18, 30; 18, 31; 18, 32	1-579	C-terminal TEV site and 10xHis tag
Sf9#9	pFastBac-1	*BamH*I, *Xho*I	33, 34	21-617	Native signal peptide & N-terminal strep tag II and TEV site
Sf9#10	pFastBac-1	*BamH*I, *Xho*I	33, 34	21-579	Native signal peptide & N-terminal strep tag II and TEV site
Sf9#11	pFastBac-1	*BamH*I, *Xho*I	18, 35; 18, 36	1-617	C-terminal TEV site and strep tag II
Sf9#12	pFastBac-1	*BamH*I, *Xho*I	18, 36	1-579	C-terminal TEV site and strep tag II

Sf9#13	pFastBac-1	*BamH*I, *Xho*I	37, 38	1-579	C-terminal $(GGGS)_2$ linker, TEV site and 10x His tag
Sf9#14	pFastBac-1	*BamH*I, *Xho*I	37, 38	1-579	C-terminal $(GGGS)_2$ linker, TEV site and strep tag II

[a] The primer sequences are listed in Table S2.

Table S2. The primers used for cloning and mutagenesis.

Primer #	Sequence
1	GCGCGGATCCATGTCTTTAAAAAAGTTT
2	CATGCTCGAGTTAACTCAATAAAGGAACTTC
3	CATGCTCGAGTTAAACTATGGAGCTCCAGTATTT
4	CATGCTCGAGTTATGGTGTACTATTTTTGAATTC
5	CATGAAGCTTTTAAACTATGGAGCTCCAGTATTTC
6	CATGAAGCTTTTAGGTGCTTGTAGAAATGTTTTC
7	CATGAAGCTTTTATGGTGTACTATTTTTGAATTC
8	GATCCTCGAGAAAACGATGGCTTTAAAAAAGTTTATAC
9	GATCCTCGAGAAAAGATTGAAAGTAATAGAAACAAG
10	GCGCTCTAGAGCACTCAATAAAGGAACTTC
11	GCGCTCTAGAGCAACTATGGAGCTCCAGTATTTC
12	GCGCTCTAGAGCTGGTGTACTATTTTTGAATTC
13	GATCGAATTCCATCATCATCATCATCATTTGAAAGTAATAGAAACAAG
14	GATCCTCGAGAAAAGAGAGGCTGAAGCTTTGAAAGTAATAGAAACAAG
15	GATCCTCGAGAAAAGAGAGGCTGAAGCTCATCATCATCATCATCATTTGAAAGTAATA GAAACAAG
16	GCGCTCTAGATTATGGTGTACTATTTTTGAACTC
17	GCGCTCTAGATTAACTCAATAAAGGAACTTC
18	GCGCGGATCCGCCACCATGTCTTTAAAAAAGTTTATAC
19	CATGCTCGAGTTAGTGGTGGTGGTGGTGGTGACTCAATAAAGGAACTTCTTC
20	CATGCTCGAGTTAGTGGTGGTGGTGGTGGTGAACTATGGAGCTCCAGTATTTC
21	ATGCTCGAGTTAGTGGTGGTGGTGGTGGTGTGGTGTACTATTTTTGAATTC
22	CATCATCATCATCATCATCATCATCATCATGAAAACCTGTATTTTCAGTCTAGATTGAA AGTAATAGAAACAAGTTATG
23	CTGTTCAAACTAATTTTGTTGTTTCCTGATATACAAAGTCATCATCATCATCATCATCA TC
24	GCGCGGATCCGCCACCATGTCTTTAAAAAAGTTTATACTGTTCAAACTAATTTTGTTG
25	CTTGTTTTTATGGTCGTATACATTTCTTACATCTATGCCGCTGCCCATCATCATCATCAT CATCATC
26	GCGCGGATCCGCCACCATGAAATTCTTAGTCAACGTTGCCCTTGTTTTTATGGTCGTAT AC
27	GTTTTATATGTGCTTTTGGCGGCGGCGGCGCATTCTGCCTTTGCGCATCATCATCATCA TCATCATC
28	GCGCGGATCCGCCACCATGGTAAGCGCTATTGTTTTATATGTGCTTTTGGCG
29	ACTGAAAGTACAGGTTCTCTGGTGTACTATTTTTGAATTC
30	TGATGATGATGATGATGAGACTGAAAGTACAGGTTCTCTG
31	TTAGTGGTGGTGGTGGTGATGATGATGATGATGAGAC
32	GCTACTCGAGTTAGTGGTGGTGGTGGTG
33	CACCCTCAGTTCGAGAAGGAAAACCTGTATTTTCAGTC
34	GGACCAGGAAGCTTTCAAACTTTGTATATCAGGAAACAAC
35	AGCAGACTGAAAGTACAGGTTCTCACTCAATAAAGGAACTTCTTC
36	CATGCTCGAGTTACTTCTCGAACTGAGGGTGGGACCAAGCAGACTGAAAGTACAGGTT C
37	GGTGGTGGTTCCGAGAACCTGTACTTTCAG
38	AGATCCTCCTCCTGGTGTACTATTTTTGAATTCTC
